# Excessive by-product formation: A key contributor to low isobutanol yields of engineered *Saccharomyces cerevisiae* strains

**DOI:** 10.1016/j.meteno.2016.01.002

**Published:** 2016-01-20

**Authors:** N. Milne, S.A. Wahl, A.J.A. van Maris, J.T. Pronk, J.M. Daran

**Affiliations:** Department of Biotechnology, Delft University of Technology, Julianalaan 67, 2628 BC Delft, The Netherlands

**Keywords:** *Saccharomyces cerevisiae*, Isobutanol, Catabolic pathway, By-product formation, 2,3-butanediol, Diacetyl

## Abstract

It is theoretically possible to engineer *Saccharomyces cerevisiae* strains in which isobutanol is the predominant catabolic product and high-yielding isobutanol-producing strains are already reported by industry. Conversely, isobutanol yields of engineered *S. cerevisiae* strains reported in the scientific literature typically remain far below 10% of the theoretical maximum. This study explores possible reasons for these suboptimal yields by a mass-balancing approach. A cytosolically located, cofactor-balanced isobutanol pathway, consisting of a mosaic of bacterial enzymes whose *in vivo* functionality was confirmed by complementation of null mutations in branched-chain amino acid metabolism, was expressed in *S. cerevisiae*. Product formation by the engineered strain was analysed in shake flasks and bioreactors. In aerobic cultures, the pathway intermediate isobutyraldehyde was oxidized to isobutyrate rather than reduced to isobutanol. Moreover, significant concentrations of the pathway intermediates 2,3-dihydroxyisovalerate and α-ketoisovalerate, as well as diacetyl and acetoin, accumulated extracellularly. While the engineered strain could not grow anaerobically, micro-aerobic cultivation resulted in isobutanol formation at a yield of 0.018±0.003 mol/mol glucose. Simultaneously, 2,3-butanediol was produced at a yield of 0.649±0.067 mol/mol glucose. These results identify massive accumulation of pathway intermediates, as well as overflow metabolites derived from acetolactate, as an important, previously underestimated contributor to the suboptimal yields of ‘academic’ isobutanol strains. The observed patterns of by-product formation is consistent with the notion that *in vivo* activity of the iron–sulphur-cluster-requiring enzyme dihydroxyacid dehydratase is a key bottleneck in the present and previously described ‘academic’ isobutanol-producing yeast strains.

## Highlights

• Catabolic isobutanol production in *S. cerevisiae* is limited by by-product formation.
• Main by-products are pathway intermediates and acetatolactate degradation products.
• *Saccharomyces cerevisiae* harbours efficient BCAA intermediates export systems.
• Iron–sulphur cluster containing dihydroxyacid dehydratase may be the main bottleneck.

## 1 Introduction

Biofuels produced from renewable feedstocks offer a promising alternative for current fossil-oil based transport fuels. In comparison with bioethanol, currently the single largest product of microbial fermentation (Weber et al*.*, 2010), isobutanol offers several advantages: i) a higher energy content, similar to that of conventional gasoline (Kolodziej and Scheib, 2012), ii) a lower volatility, resulting in lower greenhouse gas emission and iii) a lower water miscibility, which facilitates storage and distribution in existing petrochemical infrastructure and use as a pure or blended fuel in existing combustion engines (Kolodziej and Scheib, 2012). Furthermore, isobutanol can be enzymatically or chemically converted to a wide range of economically relevant compounds, including isobutyl acetate (Altiokka and Citak, 2003), *p*-xylene (Peters et al., 2010), polyisobutylene (Wettling et al., 2013), kerosene (Ilika, 2010), and polyethylene terephthalate (PET) (Kolodziej and Scheib, 2012). When produced from cellulosic biomass, isobutanol can meet the specifications required to qualify as an advanced biofuel, with an over 50% lower greenhouse gas emission than conventional gasoline (Brat and Boles, 2013, Generoso et al., 2015, Kolodziej and Scheib, 2012).

**Fig. 1:**
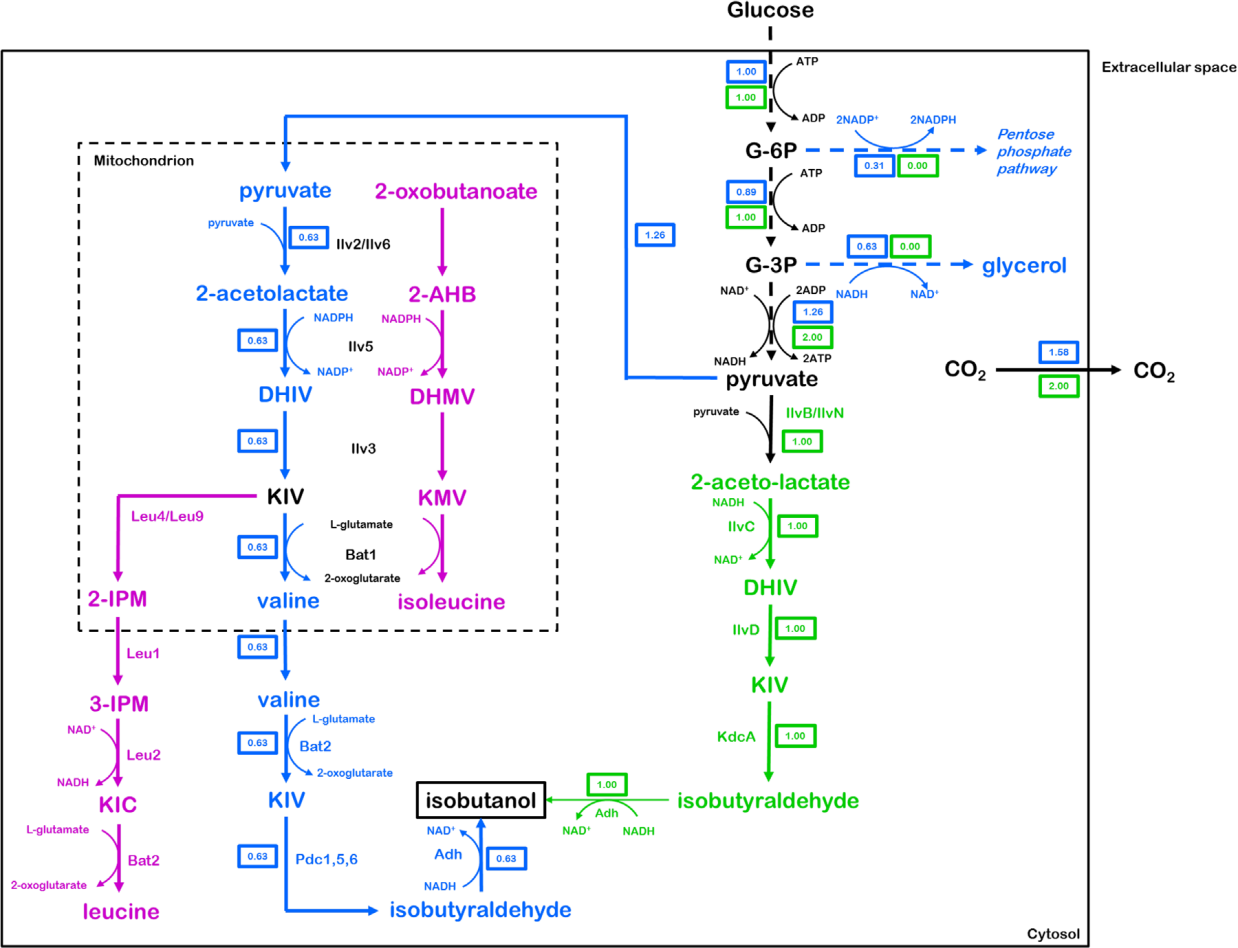
Schematic representation of branched-chain amino acid biosynthesis and isobutanol production in *S. cerevisiae.* Blue: Theoretical isobutanol production pathway using native *S. cerevisiae* reactions, with concomitant ribulose-5-phosphate production (via the oxidative pentose phosphate pathway) to regenerate NADPH consumed by Ilv5 and glycerol production to regenerate NAD^+^ consumed in lower glycolysis. Green: Redox-cofactor-balanced catabolic isobutanol production pathway with regeneration of NAD^+^ consumed in lower glycolysis by IlvC and Adh. Purple: Native pathway for the biosynthesis of leucine and isoleucine. Black: reactions common to all pathways. Dashed arrows represent multiple enzyme-catalysed reactions. Numbered boxes represent distribution of glucose flux in case of theoretically maximum product yields for the native and redox-balanced catabolic pathways (expressed in mol) as determined by stoichiometric modelling. G-6P: glucose-6-phosphate, G-3P: glyceraldehyde-3-phosphate, DHIV: 2,3-dihydroxyisovalerate, KIV: α-ketoisovalerate, 2-AHB: 2-aceto-2-hydroxybutyrate, DHMV: 2,3-dihydroxymethylvalerate, KMV: α-ketomethylvalerate, 2-IPM: 2-isopropylmalate, 3-IPM: 3-isopropylmalate, KIC: α-ketoisocaproate. (For interpretation of the references to colour in this figure legend, the reader is referred to the web version of this article.)

*Saccharomyces cerevisiae* naturally produces isobutanol as an end product of valine catabolism via the Ehrlich pathway (Ehrlich, 1907, Dickinson et al., 1998, Hazelwood et al., 2008). As this yeast can, moreover, convert pyruvate, the product of glycolysis, into valine via its mitochondrial valine biosynthesis pathway (Ryan and Kohlhaw, 1974), it contains all genetic information required for *de novo* isobutanol production from glucose (Fig. 1). However, when grown on ammonium sulphate as sole nitrogen source, tight regulation of the valine biosynthetic pathway prevents isobutanol formation (Jones and Fink, 1982, Vuralhan et al., 2005).

After many years of research, academic studies on isobutanol production by *S. cerevisiae* have generated yields that remain far below the theoretical maximum yield of 1 mol isobutanol/mol glucose (reviewed by (Generoso et al., 2015). For example, overexpression of the native *S. cerevisiae* valine biosynthesis and degradation pathways led to isobutanol yields of only 0.0059 mol/mol glucose (Chen et al., 2011), while additional elimination of competing enzymes such as Bat1, Leu2, Ald6, Ecm31, Pdc1 and Lpd1 resulted in significant but moderate increases of isobutanol yields (Ida et al., 2015, Kondo et al., 2012, Matsuda et al., 2013, Park et al., 2014). Another challenge in engineering the native yeast valine pathway is its distribution over the cytosol and mitochondria. To circumvent problems related to intracellular metabolite transport and redox co-factor balancing, two studies explored expression of complete isobutanol pathway localization into either the mitochondria (Avalos et al., 2013) or cytosol (Brat et al., 2012). The relatively small improvements in isobutanol production resulting from these strategies indicate the existence of other, significant constraints. However, a lack of mass balances and quantitative data on concentrations of pathway intermediates made it difficult to identify potential rate-controlling reactions in previously described engineered strains. While academic literature has consistently reported isobutanol yields far below the maximum theoretical yield, industrial research has already resulted in *S. cerevisiae* strains that produce isobutanol at 85% of the maximum theoretical yield (Ryan, 2015). While the cryptic nature of patent literature makes it difficult to define the exact engineering strategies, the near-theoretical yields indicate that isobutanol is produced as the main catabolic product in these strains. Akin ethanol biosynthesis under anaerobic conditions, a catabolic pathway requires a net generation of ATP, sufficient pathway flux to support cellular maintenance and growth, and efficient redox cofactor balancing without the need for external electron acceptors. With respect to the latter, the set of native *S. cerevisiae* reactions that forms the basis for previous academic studies is not in itself redox balanced due to the use of an NADPH-dependent acetohydroxyacid reductoisomerase (AHAR, encoded by *ILV5*) to catalyse the conversion of acetolactate to 2,3-dihydroxyisovalerate. Using a heterologous NADH-dependent AHAR as well as an NADH-dependent alcohol dehydrogenase offers the possibility to regenerate the NADH cofactors produced during the conversion of glucose to pyruvate (glycolysis) (Fig. 1).

This study aims to investigate the reason for the low product yields in previous academic reports on engineered, isobutanol-producing *S. cerevisiae* strains. To this end, *S. cerevisiae* was engineered to cytosolically express a redox-cofactor balanced, ATP-yielding isobutanol pathway. Subsequently, a complete analysis of the production of pathway intermediates and derived metabolites was performed in aerobic and micro-aerobic cultures. The results of this analysis were used to quantify fluxes towards isobutanol and by-products.

## 2 Materials and methods

### 2.1 Media, strains and maintenance

All *S. cerevisiae* strains used in this study (Table 1) share the CEN.PK genetic background (Entian and Kötter, 2007, Nijkamp et al., 2012). Frozen stocks of *Escherichia coli* and *S. cerevisiae* strains were prepared by addition of glycerol (30% (v/v)) to exponentially growing cells and aseptically storing 1 mL aliquots at −80 °C. Cultures were grown in synthetic medium (SM) [3 g/L KH_2_PO_4_, 0.5 g/L MgSO_4_7H_2_O and 5 g/L (NH_4_)_2_SO_4_] (Verduyn et al., 1992) with appropriate growth factors added (Pronk, 2002) and the pH adjusted to 6.0. Cultures were also grown in complex YP medium [10 g/L yeast extract, 20 g/L peptone]. Synthetic medium and complex medium with glucose as sole carbon source (SMG/YPD) contained 20 g/L glucose. Tween-80 (420 mg/L) and ergosterol (10 mg/L) were added were added to media for anaerobic cultures. Synthetic medium agar plates were prepared as described above but with the addition of 20 g/L agar (Becton Dickinson B.V. Breda, The Netherlands).

**Table 1:** *S. cerevisiae* strains used in this study.

Name	Relevant genotype	Origin
CEN.PK113-3B	MATa *ura3-52 his3-Δ1 MAL2-8c SUC2*	(Entian and Kötter, 2007)
IME169	MATa *ura3-52 his3-Δ1 MAL2-8c SUC2* pUDE189	This study
IMK463	MATa *ura3-52 his3-Δ1 MAL2-8c SUC2 ilv2::loxP-*natNT2*-loxP*	This study
IMK464	MATa *ura3-52 his3-Δ1 MAL2-8c SUC2 ilv3::loxP-*natNT2*-loxP*	This study
IMK465	MATa *ura3-52 his3-Δ1 MAL2-8c SUC2 ilv5::loxP-*natNT2*-loxP*	This study
IMK466	MATa *ura3-52 his3-Δ1 MAL2-8c SUC2 ilv6::loxP-*natNT2*-loxP*	This study
IMZ346	MATa *ura3-52 his3-Δ1 MAL2-8c SUC2 ilv2::loxP-*natNT2*-loxP* pUDE189	This study
IMZ347	MATa *ura3-52 his3-Δ1 MAL2-8c SUC2 ilv3::loxP-*natNT2*-loxP* pUDE189	This study
IMZ348	MATa *ura3-52 his3-Δ1 MAL2-8c SUC2 ilv5::loxP-*natNT2*-loxP* pUDE189	This study
IMZ349	MATa *ura3-52 his3-Δ1 MAL2-8c SUC2 ilv6::loxP-*natNT2*-loxP* pUDE189	This study
IMZ500	MATa *ura3-52 HIS3 MAL2-8c SUC2 pdc1::loxP pdc5::loxP pdc6::loxP MTH1∆T* p426GPD	This study
IMI302	MATa *ura3-52 HIS3 MAL2-8c SUC2 pdc1::loxP pdc5::loxP pdc6::cas9-tagA-loxP-*natNT2*-loxP MTH1∆T ade2::PDC1-amdS*	(Milne et al., 2015)
IMX708	MATa *ura3-52 HIS3 MAL2-8c SUC2 pdc1::loxP pdc5::loxP pdc6::cas9-tagA-loxP-*natNT2*-loxP MTH1∆T ilv2Δ::*^*co*^*ilvB* hphNT1^*co*^*ilvC*^*6E6 co*^*ilvD*^*co*^*ilvN*^*M13*^	This study
IME305	MATa *ura3-52 HIS3 MAL2-8c SUC2 pdc1::loxP pdc5::loxP pdc6::cas9-tagA-loxP-*natNT2*-loxP MTH1∆T ilv2Δ::*^*co*^*ilvB* hphNT1 ^*co*^*ilvC*^*6E6 co*^*ilvD*^*co*^*ilvN*^*M13*^ p426-GPD	This study
IME306	MATa *ura3-52 HIS3 MAL2-8c SUC2 pdc1::loxP pdc5::loxP pdc6::cas9-tagA-loxP-*natNT2*-loxP MTH1∆T ilv2Δ::*^*co*^*ilvB* hphNT1 ^*co*^*ilvC*^*6E6 co*^*ilvD*^*co*^*ilvN*^*M13*^ pUDE001	This study
IME307	MATa *ura3-52 HIS3 MAL2-8c SUC2 pdc1::loxP pdc5::loxP pdc6::cas9-tagA-loxP-*natNT2*-loxP MTH1∆T ilv2Δ::*^*co*^*ilvB* hphNT1 ^*co*^*ilvC*^*6E6 co*^*ilvD*^*co*^*ilvN*^*M13*^ pUDE321	This study
IME308	MATa *ura3-52 HIS3 MAL2-8c SUC2 pdc1::loxP pdc5::loxP pdc6::cas9-tagA-loxP-*natNT2*-loxP MTH1∆T ilv2Δ::*^*co*^*ilvB* hphNT1 ^*co*^*ilvC*^*6E6 co*^*ilvD*^*co*^*ilvN*^*M13*^ pUDE336	This study

### 2.2 Strain and plasmid construction

#### 2.2.1 Expression cassettes for isobutanol biosynthetic genes

DNA coding sequences of *Corynebacterium glutamicum ilvN*^M13^ and *C. glutamicum ilvB* (Elisakova et al., 2005), *E. coli ilvC*^6E6^ (Bastian *et al.*, 2011) and *Lactococcus lactis ilvD* (Urano et al., 2012) were codon optimised for *S. cerevisiae* using the JCat algorithm (Grote et al., 2005) (Supplementary materials). Custom synthesized cassettes cloned into pUC57 (Y14837.1) were provided by BaseClear (Leiden, The Netherlands). In these vectors, the codon optimized genes (^co^) were flanked by strong constitutive promoters and terminators from *S. cerevisiae* glycolytic genes. Each cassette was further flanked with 60 bp tags (labelled A through I) with homology to an adjacent cassette. These tags have no significant homology to the *S. cerevisiae* genome, ensuring that each cassette can only recombine with an adjacent cassette using homologous recombination (Kuijpers et al., 2013). Custom synthesis resulted in plasmids pUD220 (D-*TEF1*_*P*_*-*^*co*^*ilvN*^*M13*^*-CYC1*_*t*_-C), pUD221 (B-*TPI1*_*P*_*-*^*co*^*ilvB-ADH1*_*t*_-C), pUD222 (D-*ADH1*_*P*_*-*^*co*^*ilvC*^*6E6*^*-PYK1*_*t*_-F) and pUD223 (G-*PGK1*_*P*_*-*^*co*^*ilvD-TEF1*_*t*_-I). Each plasmid was transformed into chemically competent *E. coli* (T3001, Zymo Research, Irvine, CA) according to the manufacturer’s instructions, and the gene sequences confirmed by Sanger sequencing (BaseClear). The gene cassettes from each plasmid were used to assemble the plasmid pUDE189, in association with cassettes encoding a *URA3* yeast selection marker (pUD192: A-*URA3*-B), a *CEN6-ARS4* yeast replicon (pUD193: F-*CEN6-ARS4*-G), and a fragment containing the *bla* (AmpR) ampicillin resistance marker and *E. coli* origin of replication (pUD195: I-AmpR-A) to allow selection and propagation in both *S. cerevisiae* and *E. coli* (Kozak et al., 2014b) (Table 2). Plasmids propagated in *E. coli* were isolated with Sigma GenElute Plasmid Kit (Sigma Aldrich, Zwijndrecht, The Netherlands). Each cassette was flanked by unique restriction sites allowing them to be excised from the plasmid backbone to generate fragments to use in the assembly of pUDE189 by vector assembly via homologous recombination. For digestion of each plasmid, high fidelity restriction endonucleases (Thermo Scientific, Waltham, MA) were used according to the manufacturer’s instructions. pUD220, pUD222 and pUD223 were digested with ApaI and BamHI, pUDE221 was digested with XmnI and BamHI, pUD192 was digested with XhoI, pUD194 was digested with SacII and pUD195 was digested with NotI. After digestion, each fragment was purified by gel electrophoresis using 1% (w/v) agarose (Sigma Aldrich) in TAE buffer (40 mM Tris-acetate pH 8.0 and 1 mM EDTA). Isolation of agarose trapped DNA fragments was performed using Zymoclean Gel DNA Recovery Kit (Zymo Research). Equimolar amounts of each fragment were transformed into CEN.PK113-3B (*ura3-52*, *his3-Δ1*) allowing for *in vivo* vector assembly of the fragments by homologous recombination. Correctly assembled transformants were selected on SMG agar supplemented with histidine (0.125 g/L). A single colony isolate was stocked as IME166 (Table 1). Correct plasmid assembly was verified using primer pairs which bound in each of the gene cassettes and amplified the 60 bp homologous tags (Table 3). The plasmid was extracted from IME166, named as pUDE189 and transformed into *E. coli* DH5α by electroporation in 2 mM cuvettes (BioRad, Hercules, CA) using a Gene PulserXcell electroporation system (BioRad) following the manufacturer’s protocol and stocked in the *E. coli* host.

**Table 2:** Plasmids used in this study

Name	Characteristics	Origin
pUC57	*bla (Ap*^*R*^*), rep (pMB1 E. coli* replicon) (NCBI accession number: Y14837.1)	BaseClear
pUD192	pUC57+*URA3*	(Kozak et al., 2014b)
pUD194	pUC57+2 µm replicon	(Kozak et al., 2014b)
pUD195	*bla (Ap*^*R*^*), rep (pMB1*)	(Kozak et al., 2014a)
pUD220	pUC57+*TEF1*_*P*_*-*^co^*ilvN*^*M13*^*-CYC1*_*t*_	This study
pUD221	pUC57+*TPI1*_*P*_*-*^co^*ilvB-ADH1*_*t*_	This study
pUD222	pUC57+*ADH1*_*P*_*-*^co^*ilvC*^*6E6*^*-PYK1*_*t*_	This study
pUD223	pUC57+*PGK1*_*P*_*-*^co^*ilvD-TEF1*_*t*_	This study
pUDE189	2µm ori, *bla (Ap*^*R*^*), URA3 TEF1*_*P*_*-*^co^*ilvN*^*M13*^*-CYC1*_*t*_*TPI1*_*P*_*-*^co^*ilvB-ADH1*_*t*_*, ADH1*_*P*_*-*^co^*ilvC*^*6E6*^*-PYK1t PGK1*_*P*_*-*^co^*ilvD-TEF1*_*t*_	This study
p426GPD	2µm ori, *URA3 TDH3*_*P*_*-CYC1t*	(Mumberg et al., 1995)
pUDE001	2µm ori, *URA3 TDH3*_*P*_*-ARO10-CYC1*_*t*_	(Vuralhan et al., 2005)
pUDE321	2µm ori, *URA3 TDH3*_*P*_*-*^co^*kdcA-CYC1*_*t*_	(Milne et al., 2015)
pUDE336	2µm ori, *URA3 TDH3*_*P*_*-*^co^*kivD-CYC1*_*t*_	(Milne et al., 2015)
pUGnatNT2	2µm ori, *URA3 TEF2*_*P*_*-*natNT1*-TEF2*_*t*_	(de Kok et al., 2012)

^co^: Codon optimised.

**Table 3:** Oligonucleotide primers used in this study

Name	Sequence (5′→3′)
Plasmid/integration conformation	
A-tag amp fwd	AAATAAACAAATAGGGGTTCCGC
A-tag amp rev	GAAATGCTGGATGGGAAGCG
B-tag amp fwd	TCCCATATGATTGTCTCCGTAAGCTCG
B-tag amp rev	ACTCTGTCATATACATCTGCCGCAC
C-tag amp fwd	GCAAATGCCTGCAAATCG
C-tag amp rev	CGCGTGTACGCATGTAAC
D-tag amp fwd	GCTAAATGTACGGGCGACAG
D-tag amp rev	GCCTTCATGCTCCTTGATTTCC
F-tag amp fwd	GTCGTCATAACGATGAGGTGTTGC
F-tag amp rev	ATGAAGCACAGATTCTTCGTTG
G-tag amp fwd	GAGAAGAACGGCATAGTGCGTG
G-tag amp rev	GTAAGTTTCACGAGGTTCTAC
I-tag amp fwd	GCGTCAATCGTATGTGAATGC
I-tag amp rev	GCCTTTGAGTGAGCTGATACC
ILV2 upstream fwd	TCCTTTCTCCACCATCCCTA
ILV2 downstream rev	CGTGTCCGACGAGTTAAAAC
Knockout cassette amplification	
ILV2 KO fwd	TTTACAAAATCTAAACCCTTTGAGCTAAGAGGAGATAAATACAACAGAATCAATTTTCAACAGCTGAAGCTTCGTACGC
ILV2 KO rev	AATAATAATAAAGTCTGCATTTTTTACTGAAAATGCTTTTGAAATAAATGTTTTTGAAATGCATAGGCCACTAGTGGATCTG
ILV3 KO fwd	CTGTAATCTTTAGTAACGGATTCTTGTATTTTTTTGTAAACAGCCAAGAAAAAAGTAGAGCAGCTGAAGCTTCGTACGC
ILV3 KO rev	AAAGATGATGGAAAAGGAGAATCTCTATATATATATTCATCGATTGGGGCCTATAATGCAGCATAGGCCACTAGTGGATCTG
ILV5 KO fwd	AACCTATTCCTAGGAGTTATATTTTTTTACCCTACCAGCAATATAAGTAAAAAATAAAACCAGCTGAAGCTTCGTACGC
ILV5 KO rev	ACTTGATGTTGCAAAAATTCCAAGAGAAAAAGTTTCCAGCACTTGATATTATTTTCCTCTGCATAGGCCACTAGTGGATCTG
ILV6 KO fwd	TACATAGTTCGTATATACAGAATCTTTAGAACATCTGAGCTCACTAACCCAGTCTTTCTACAGCTGAAGCTTCGTACGC
ILV6 KO rev	TACGTTATATAGATGTATAGAGGAGAGTCCCGAGGGCGATCGCAAGGCCGAGAGACTAACGCATAGGCCACTAGTGGATCTG
Knockout conformation	
ILV2 upstream fwd	TCCTTTCTCCACCATCCCTA
ILV2 downstream rev	CGTGTCCGACGAGTTAAAAC
ILV3 upstream fwd	CCCTCTTGTATCCATTCC
ILV3 downstream rev	CTTTAGTGGCAGCAAAGC
ILV5 upstream fwd	GTTGTGCGCGTGCACATTTC
ILV5 downstream rev	AATCGTAGCTGTCCCGATGAGG
ILV6 upstream fwd	GCACATCCAACGAATCACCTCACCGTTATC
ILV6 downstream rev	CGCGTCACCTCGTACAAACGTACAATC
Verification of plasmid transformation	
GPD1 promoter Fwd	GGGATGTGCTGCAAGGCGATTAAGTTGG
CYC1 terminator Rev	GGCAGTGAGCGCAACGCAATTAATGTGAG
Cassette integration	
ilvD amp with ILV2 hom fwd	TTTACAAAATCTAAACCCTTTGAGCTAAGAGGAGATAAATACAACAGAATCAATTTTCAAGCCAGAGGTATAGACATAGCCAGAC
ilvD amp (I-tag rev)	AGACGTCGCGGTGAGTTCAG
hphNT1 amp with I-tag hom fwd	TATTCACGTAGACGGATAGGTATAGCCAGACATCAGCAGCATACTTCGGGAACCGTAGGCCCAGCTGAAGCTTCGTACGC
hphNT1 amp with B-tag hom rev	GTTGAACATTCTTAGGCTGGTCGAATCATTTAGACACGGGCATCGTCCTCTCGAAAGGTGGCATAGGCCACTAGTGGATCTG
ilvB amp (B-tag fwd)	TACTCGCCGATAGTGGAAAC
ilvB amp (C-tag rev)	CGCGTGTACGCATGTAAC
ilvN amp (C-tag fwd)	GCAAATGCCTGCAAATCG
ilvN amp (D-tag rev)	GCCTTCATGCTCCTTGATTTCC
ilvC amp (D-tag fwd)	GCTAAATGTACGGGCGACAG
ilvC amp with ILV2 hom rev	AATAATAATAAAGTCTGCATTTTTTACTGAAAATGCTTTTGAAATAAATGTTTTTGAAATTGCCGAACTTTCCCTGTATGAAGC

#### 2.2.2 Branched-chain amino acid pathway gene deletions

*ILV2*, *ILV3*, *ILV5* and *ILV6* deletion cassettes were constructed by amplifying the natNT2 cassette from pUGnatNT2 (de Kok et al., 2012) using primers with added homology to the upstream and downstream region of each respective gene (Table 3). Each individual natNT2 deletion cassette was transformed into CEN.PK113-3B (*ura3-52*, *his3-Δ1*) yielding strains IMK463 (*ilv2Δ*), IMK464 (*ilv3Δ*), IMK465 (*ilv5Δ*) and IMK466 (*ilv6Δ*). Transformants were selected on complex medium agar (YPD) supplemented with 100 mg/L nourseothricin (Jena Bioscience, Jena, Germany). Each strain was then transformed with pUDE189 yielding strains IMZ346 (*ilv2Δ*, pUDE189), IMZ347 (*ilv3Δ*, pUDE189), IMZ348 (*ilv5Δ,* pUDE189) and IMZ349 (*ilv6Δ*, pUDE189) (Table 1). Transformants were selected on SMG agar supplemented with histidine (0.125 g/L) and nourseothricin (100 mg/L).

#### 2.2.3 Construction of heterologous pathway strains

*S. cerevisiae* IMX708 was constructed by integrating the ^*co*^*ilvB*, ^*co*^*ilvC*^*6E6*^, ^*co*^*ilvD* and ^*co*^*ilvN*^*M*^*13*^^ overexpression cassettes along with a hphNT1 dominant selection marker conferring resistance to hygromycin (Goldstein and McCusker, 1999) at the *ILV2* locus of IMI302 (Milne et al., 2015) using the CRISPR-Cas system (Mans et al., 2015). Cassettes were amplified by PCR using primers which either bound in the already introduced 60bp tags of each cassette, or primers with added homology to an adjacent 60 bp cassette or to the flanking regions of the *ILV2* locus in order to allow *in vivo* assembly of adjacent cassettes and subsequent integration. The ^*co*^*ilvD* cassette was amplified from pUD223 with a primer that introduced homology to the upstream *ILV2* region and a primer which bound in the 60 bp I-tag already flanking the cassette (ilvD amp with ILV2 hom fwd/ ilvD amp (I-tag rev)). The ^*co*^*ilvC*^*6E6*^ cassette was amplified from pUD222 with a primer which bound in the 60 bp D-tag already flanking the cassette and a primer that introduced homology to the downstream *ILV2* region (ilvC amp (D-tag fwd)/ ilvC amp with ILV2 hom rev). The ^*co*^*ilvB* cassette was amplified from pUD221 with primers that bound in the B and C tags already flanking the cassette (ilvB amp (B-tag fwd)/ ilvB amp (C-tag rev)). The ^*co*^*ilvN*^*M13*^ cassette was amplified from pUD220 using primers which annealed in the C and D tags already flanking the cassette (ilvN amp (C-tag fwd)/ ilvN amp (D-tag rev)). Finally the *hphNT1* cassette was amplified from pUGhphNT1 with primers that introduced homology to the I and B tags (hphNT1 amp with I-tag hom fwd/ hphNT1 amp with B-tag hom rev). Targeted integration of these cassettes at the *ILV2* locus was facilitated by the CRISPR-Cas system according to the *in vivo* plasmid assembly protocol described by (Mans et al., 2015). Assembly of the required plasmid containing the *ILV2* specific guide RNA and subsequent Cas9 mediated removal of the *ILV2* gene was achieved in a single *in vivo* homologous recombination reaction step. Transformation of the CRISPR plasmid backbone, the *ILV2* specific guide RNA fragment and the homologously linked expression cassettes resulted in the *in vivo* assembly of the plasmid, a Cas9 mediated double strand break in the *ILV2* gene, and repair of that break using the homologously assembled expression cassettes with homology to the upstream and downstream regions of *ILV2* (Fig. 3). Correctly assembled transformants were first selected on SMG agar plates supplemented 0.5 g/L valine, leucine and isoleucine as well as 200 mg/L hygromycin and in the absence of adenine supplementation to induce the loss of the transient *PDC1* cassette (Milne et al., 2015). Single colonies were then streaked 3 times onto SMG agar plates containing 200 mg/L hygromycin, 1 g/L 5-fluoorotic acid (5’FOA) and 0.150 g/L uracil to induce the loss of the *ILV2* targeting *in vivo* assembled CRISPR plasmid, without valine, leucine and isoleucine supplementation. A single colony isolate with restored branched chain amino acid biosynthesis was stocked and labelled as IMX708. The uracil auxotrophy of this strain was then complemented by transformation with p426GPD and pUDE321 resulting in strains IME305 (*URA3*), and IME307 (^*co*^*kdcA URA3*) respectively. The Pdc^-^ control strain IMZ500 was constructed by transforming IMI244 with the p426GPD (*URA3*) plasmid.

In all cases PCR amplification of the gene cassettes was performed using Phusion® Hot Start II High Fidelity Polymerase (Thermo scientific) according to the manufactures instructions using HPLC or PAGE purified, custom synthesized oligonucleotide primers (Sigma Aldrich) in a Biometra TGradient Thermocycler (Biometra, Gottingen, Germany). Conformation of plasmid assembly/transformation, gene knockout and genome integration was achieved using the diagnostic primers listed in Table using DreamTaq (Thermo scientific) and desalted primers (Sigma Aldrich) in a Biometra TGradient Thermocycler (Biometra).

### 2.3 Shake flask cultivation, bioreactor-batch fermentation and micro-aerobic high cell density cultivation

All *S. cerevisiae* strains were grown in complex medium (YPD) or synthetic medium (SMG) (Verduyn et al., 1990) containing 20 g/L glucose. If required, 125 mg/L histidine and/or 150mg/L uracil was added to the synthetic media in order to complement a histidine and/or uracil auxotrophy. Cultures were grown in either 250 mL or 500 mL shake flasks containing 50 mL or 100 mL medium with incubation at 30 °C in an Innova incubator shaker (New Brunswick Scientific, Edison, NJ) at 200 rpm. Optical density at 660 nm was measured using a Libra S11 spectrophotometer (Biochrom, Cambridge, United Kingdom).

Controlled aerobic batch cultivation was carried out at 30 °C in 2 L bioreactors (Applikon, Schiedam, The Netherlands) with a working volume of 1 L. Synthetic medium was supplemented with 20 g/L glucose and 0.2 g/L of Pluronic antifoam (BASF, Ludwigshaven, Germany). The pH was kept constant at pH 5.0 by automatic addition of 2 M KOH. The stirrer speed was constant at 800 rpm and the aeration rate kept at 500 mL/min.

Micro-aerobic high-cell-density cultures were studied in SMG medium supplemented with Tween-80 (420 mg/L) and ergosterol (10 mg/L) in a total volume of 25 mL in 30 mL rubber stopper serum bottles. In contrast to pH control batch fermentation and to prevent a too fast acidification of the culture medium the initial pH was set to 6.0.with 2 M KOH. High-cell-density cultures were prepared by growing each strain in a 1 L aerobic batch fermentation setup. Cell cultures were harvested then centrifuged at 4700 g for 5 min then resuspended to a final OD660 of ~50. After inoculation into 30 mL serum stopper bottles the cap was tightly sealed to create micro-aerobic conditions. Rubber stoppers were pierced with a 0.6 mM Microlance needle (Becton Dickinson) to prevent pressure build-up. Each needle head also contained a cotton plug to prevent contamination. Cultures were incubated at 30 °C. Samples were taken to determine extracellular metabolite concentrations, OD660 and pH over the linear phase of glucose consumption. To limit the introduction of oxygen into the cultures during sampling, liquid samples were taken by attaching a sterile syringe to the pierced needle, inverting the serum bottle and withdrawing ~200 µL. The biomass concentration of each culture was estimated by taking the average OD 660 value and assuming 1 g/L of cell biomass equates to an OD660 value of 4.02.

### 2.4 Analytical methods

Biomass dry weight from bioreactors was determined by filtration of 10 mL broth over pre-dried and weighed 0.45 µm nitrocellulose filters (Gelman Laboratory, Ann Arbor, MI). After filtration the filters were dried for 20 min in a microwave at 350 W. To determine general extracellular metabolite concentrations, culture samples were spun down at 3500 g and the supernatant was collected. Metabolites were analysed using an Agilent 1260 Affinity HPLC machine (Agilent Technologies, Amstelveen, The Netherlands) with an Aminex HPX-87H ion exchange column (BioRad) operated at 60 °C with a mobile phase of 5 mM H_2_SO_4_ and a flow rate of 0.6 mL/min. Extracellular diacetyl was determined using static headspace gas chromatography. 5 mL of supernatant sample with 20 mg/L 2,3-hexandione as internal standard was heated to 65 °C for 30 min prior to injection using a CTC Combi Pal headspace autoinjector (CTC Analytics AG, Zwingen, Switzerland). Samples were analysed using a 7890A Agilent GC (Agilent Technologies) with an electron capture detector on a CP-Sil 8 CB (50 m×530 µm×1 µm) capillary column (Agilent Technologies). The split ratio was 1:1 with a split flow of 8 mL nitrogen per minute. The injector was set at 120 °C and an oven temperature profile of 35 °C for 3 min followed by an increase of 10 °C/min to 95 °C was used. The ECD detector was set at 150 °C was a make-up flow of 10 mL/min of nitrogen gas.

Samples for intracellular metabolite measurements were collected in pre-weighed tubes containing 30 mL 100% methanol kept at −40 °C. Approximately 6 mL of broth (~2 mg biomass) were quenched in methanol and the tubes weighed again to determine the exact volume added and vortexed. The samples were then centrifuged for 5 min at 10,000 g at −19 °C. The supernatant was discarded and the cell pellet was resuspended in 6 mL 100% methanol, and centrifuged again for 5 min at 10,000 g at −19 °C. The supernatant was discarded and 120 µL of ^13^C cell extract (as internal standard) was added to the cell pellet and the mix was resuspended in 2.5 mL pre-cooled 50% (v/v) aqueous methanol and 2.5 mL pre-cooled 100% chloroform. Samples were vigorously shaken for 45 min in an orbital shaker using a custom-made tube adaptor at −40 °C. Samples were then centrifuged for 5 min at 5000 g at −19 °C. The resulting upper layer (water/methanol) containing the metabolites of interest was transferred. To transfer putative remainders in the chloroform phase, the extraction was repeated by adding 2.5 mL water/methanol to the remaining chloroform layer. Excess liquid was removed using the Rapidvap system (Labconco, Kansas city, MO) and the dried samples were resuspended in 600 µL MilliQ water and stored until analysis at −80 °C. Samples for extracellular amino acid determination were prepared by passing broth through a filter and collecting the filtrate. The amino acid concentrations were determined using the *N-*Methyl-*N*-*tert*-butyldimethylsilytrifluoroacetamide (MTBSTFA) derivatization method according to (Dauner and Sauer, 2000) using 100 µL of intracellular sample or 10 µL of extracellular sample.

### 2.5 Stoichiometric modelling and metabolic flux analysis

The metabolic model was set up based on the pathway stoichiometries from MetaCyc (Caspi et al., 2008). To obtain a compact model, linear reactions were lumped. The lumped reactions included were glycolysis (simplified), the pentose-phosphate pathway and the TCA cycle (included as a single mitochondrial localized reaction). Furthermore, the electron transport chain and oxidative phosphorylation were included to represent and estimate a putative oxygen consumption rate. With different compartments, transporters and carriers have a major influence on the network functionality. To account for this, a lumped exchange reaction for NADPH/NADP, derived from the assumption of an active citrate/α-ketoglutarate shuttle together with NADP-dependent isocitrate-dehydrogenase and transport of the two acids was included. Also included was a lumped reaction for the exchange of NAD/NADH based on a malate/aspartate shuttle working together with aspartate transaminase and malate dehydrogenase (Palmieri et al., 2006). Additionally, a glutamate/α-ketoglutarate shuttle and valine transporter were included, as well as pyruvate transport via mitochondrial pyruvate carriers (MPC) (Herzig et al., 2012). A complete list of the metabolic network reactions can be found in Supplementary material. With this reaction network, “wild-type” and catabolic variants of the isobutanol pathway were included and the resultant metabolic flux and maximum yield determined using the software CellNetAnalyzer 2015.1 (Klamt et al., 2007). The flux map of the networks was created using Omix (Droste et al*.*, 2013) (Supplementary material).

For the theoretical yield, the isobutanol production flux was set as only target and the glucose uptake rate was set to 100. For the estimation of intracellular fluxes based on experimental data the respective genotype was taken into account (i.e. knock out of *ILV2* encoding the native mitochondrial Ilv2). The experimental standard deviation was used to weight the single measurements and the resulting flux map created using Omix (Supplementary material).

## 3 Results

### 3.1 Design of a catabolic route to isobutanol

Due to non-matching redox-cofactor specificities, a pathway that solely consists of native *S. cerevisiae* enzymes cannot support anaerobic isobutanol formation without the need for concomitant glycerol production. This redox issue limits the theoretical maximum yield of such a pathway to 0.63 mol/mol glucose and, moreover, imposes a requirement for aerobic respiration to supply ATP for cellular maintenance and growth. Production of isobutanol as sole catabolic product, with a maximum theoretical yield of 1 mol/mol glucose, requires several genetic modifications (Fig. 1). In this study, design of a catabolic isobutanol pathway was based on the following genetic interventions: 1) inactivation of the native alcoholic fermentation pathway by deletion of the pyruvate-decarboxylase genes *PDC1*, *PDC5* and *PDC6* and introduction of an internal deletion in *MTH1* to restore growth on glucose (Oud et al., 2012, van Maris et al., 2004a); 2) introduction of a cytosolic isobutanol pathway comprising (i) a feedback-insensitive regulatory subunit (IlvN^M13^) (Elisakova et al., 2005) and catalytic subunit (IlvB) (Cordes et al., 1992) of *Corynebacterium glutamicum* acetolactate synthase; (ii) an *E. coli* acetohydroxyacid reductoisomerase (EC 1.1.1.86) engineered for use of NADH as redox cofactor (IlvC^6E6^) (Bastian et al., 2011, Holmberg and Petersen, 1988); (iii) a dihydroxyacid dehydratase (EC 4.2.1.9) from *L. lactis* (IlvD), previously shown to be active in the *S. cerevisiae* cytosol (Urano et al*.*, 2012); (iv) a 2-oxo acid decarboxylase from *L. lactis* (KdcA) with a high specificity and activity towards α-ketoisovalerate upon expression in *S. cerevisiae* (Milne et al., 2015); and (v) endogenous *S. cerevisiae* NADH-dependent alcohol dehydrogenase(s) Adh2 with affinity towards isobutyraldehyde (Brat et al*.*, 2012). Provided that a sufficiently high flux through this cytosolic, redox-cofactor-balanced and ATP-yielding pathway (Fig. 1) can be achieved *in vivo*, it should allow for formation of isobutanol as sole catabolic product in anaerobic cultures.

### 3.2 In vivo activity of a heterologous branched-chain amino-acid pathway in *S. cerevisiae*

**Fig. 2:**
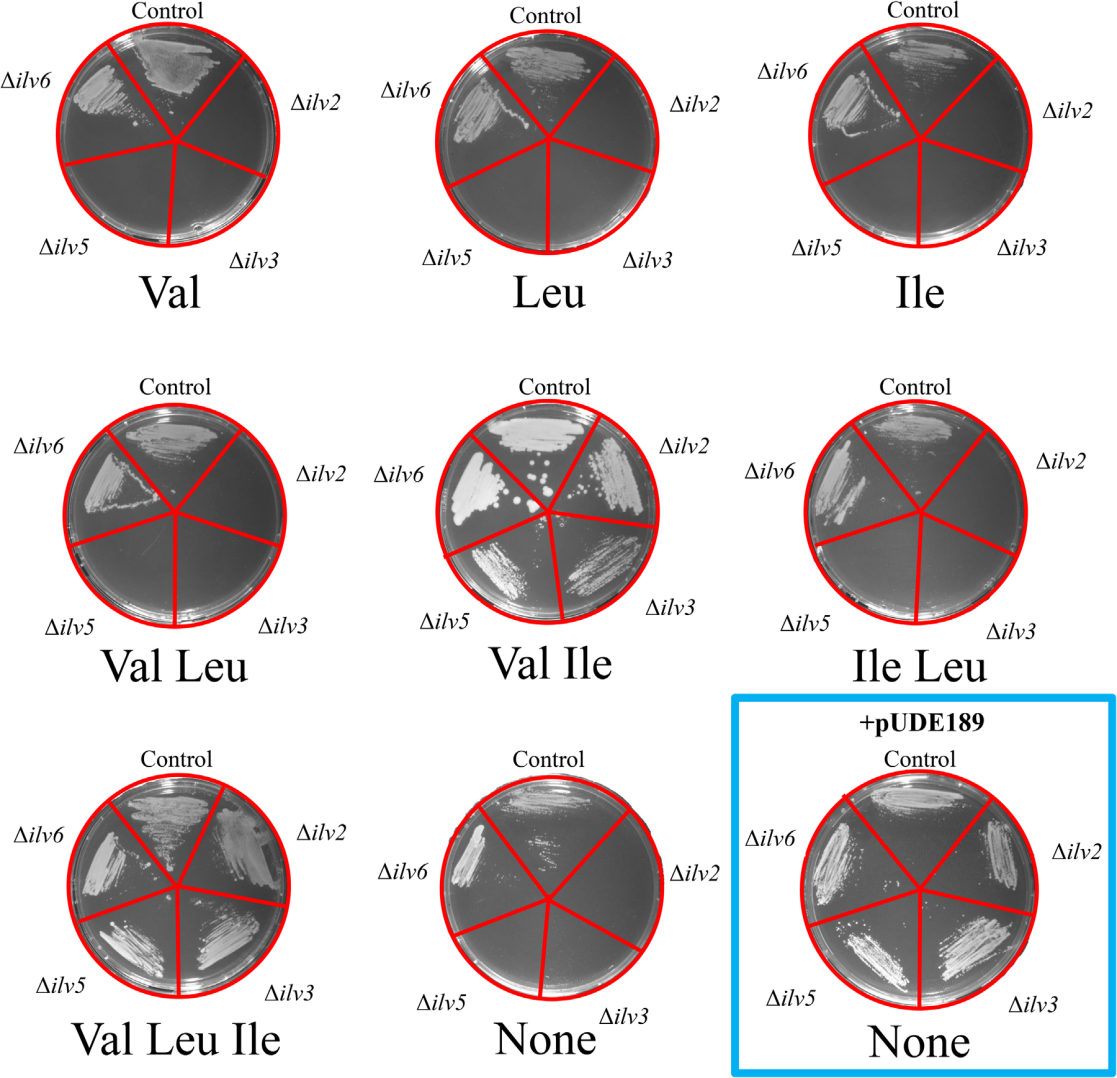
Complementation of *S. cerevisiae* deletion mutants affected in branched chain-amino acid biosynthesis with a heterologous pathway. Strains CEN.PK113-3B (control), IMK463 (Δ*ilv2*), IMK464 (Δ*ilv3*), IMK465 (Δ*ilv5*), IMK466 (Δ*ilv6*) were grown in SMG medium supplemented with histidine (0.150 g/L) and uracil (0.125 g/L). The corresponding strains complemented with the heterologous branched chain amino acid biosynthesis pathway IME169 (control+pUDE189) IMZ346 (Δ*ilv2*+pUDE189), IMZ347 (Δ*ilv3*+pUDE189), IMZ348 (Δ*ilv5*+pUDE189) and IMZ349 (Δ*ilv6*) were grown in SMG medium supplemented with histidine. Cells were then washed with water and streaked onto SMG agar plates supplemented with histidine and uracil (if required) and 5 g/L of valine (Val), leucine (Leu) and/or isoleucine (Ile) as indicated. Plates were incubated at 30 °C for 3 days.

*In vivo* activity of the heterologous enzymes involved in the conversion of pyruvate to α-ketoisovalerate (KIV) via the pathway design described above was tested by complementation of *S. cerevisiae* mutants lacking key enzymes in the native branched-chain amino-acid biosynthesis pathway (Fig. 1). Consistent with earlier studies (Kingsbury and McCusker, 2010, Velasco et al., 1993, Zelenayatroitskaya et al., 1995) strains containing deletions of individual ‘catalytic’ genes IMK463 (*ilv2*Δ), IMK464 (*ilv3*Δ), IMK465 (*ilv5*Δ) did not grow on media lacking both valine and isoleucine (Fig. 2). In these strains, the presence of valine is sufficient to restore leucine synthesis since KIV formed by transamination of valine can feed leucine biosynthesis (Fig. 1). Deletion of *ILV6* (strain IMK466) did not lead to auxotrophy (Fig. 2) due to its non-essential role as regulatory subunit of acetolactate synthase (Cullin et al., 1996, Pang and Duggleby, 1999). These single deletion mutants were transformed with plasmid pUDE189, carrying the heterologous ^*co*^*ilvB,* ^*co*^*ilvN*^*M*^*13*^^ (*C. glutamicum*), ^*co*^*ilvC*^*6E6*^ (*E. coli*), and ^*co*^*ilvD* (*L. lactis*) genes under the control of strong constitutive promoters. The resulting strains IMZ346 (*ilv2*Δ pUDE189), IMZ347 (*ilv3*Δ pUDE189), IMZ348 (*ilv5*Δ pUDE189), and IMZ349 (*ilv6*Δ pUDE189) readily grew on synthetic medium without branched-chain amino acid supplementation (Fig. 2), thereby demonstrating functional replacement of the native, mitochondrial yeast enzymes by their cytosolically expressed heterologous orthologs.

**Fig. 3:**
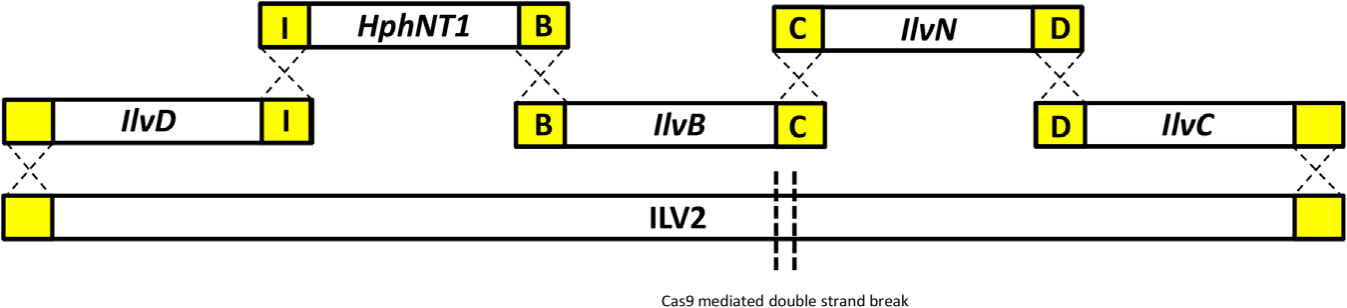
Construction and assembly of a heterologous KIV biosynthesis pathway in *S. cerevisiae* using CRISPR-Cas guided *ILV2* gene disruption and integration of the heterologous gene cassettes via homologous combination with 60 bp overlapping tags. A specific guide RNA was used to target *cas9* to *ILV2*. The resulting double-strand break at the *ILV2* locus was then repaired by the assembly and integration, by *in vivo* homologous recombination, of the expression cassettes for the codon-optimized heterologous genes that together formed the new KIV biosynthesis pathway.

To further investigate *in vivo* activity of the engineered pathway, ^*co*^*ilvB,* ^co^*ilvN*^*M*^*13*^^, ^*co*^*ilvC*^*6E6*^ and ^*co*^*ilvD* gene cassettes were integrated at the *ILV2* locus of strain IMI302 (Fig. 3), which carries a triple *PDC* deletion, combined with an *MTH1* internal deletion, to eliminate unwanted ethanol formation and allow growth on glucose (Oud et al., 2012). The resulting strain IMX708 (Δ*pdc1,5,6 Δilv2 MTH1ΔT* ^*co*^*ilvN*^*M*^*13*^^*,* ^*co*^*ilvB*, ^*co*^*ilvC*^*6E6*^, ^*co*^*ilvD*, *ura3-52*) was subsequently transformed with the p426GPD plasmid to obtain the uracil prototrophic strain IME305 (Δ*pdc156 Δilv2 MTH1ΔT* ^*co*^*ilvN*^*M*^*13*^^^*co*^*ilvB* ^*co*^*ilvC*^*6E6* co^*ilvD* p426GPD).

**Fig. 4:**
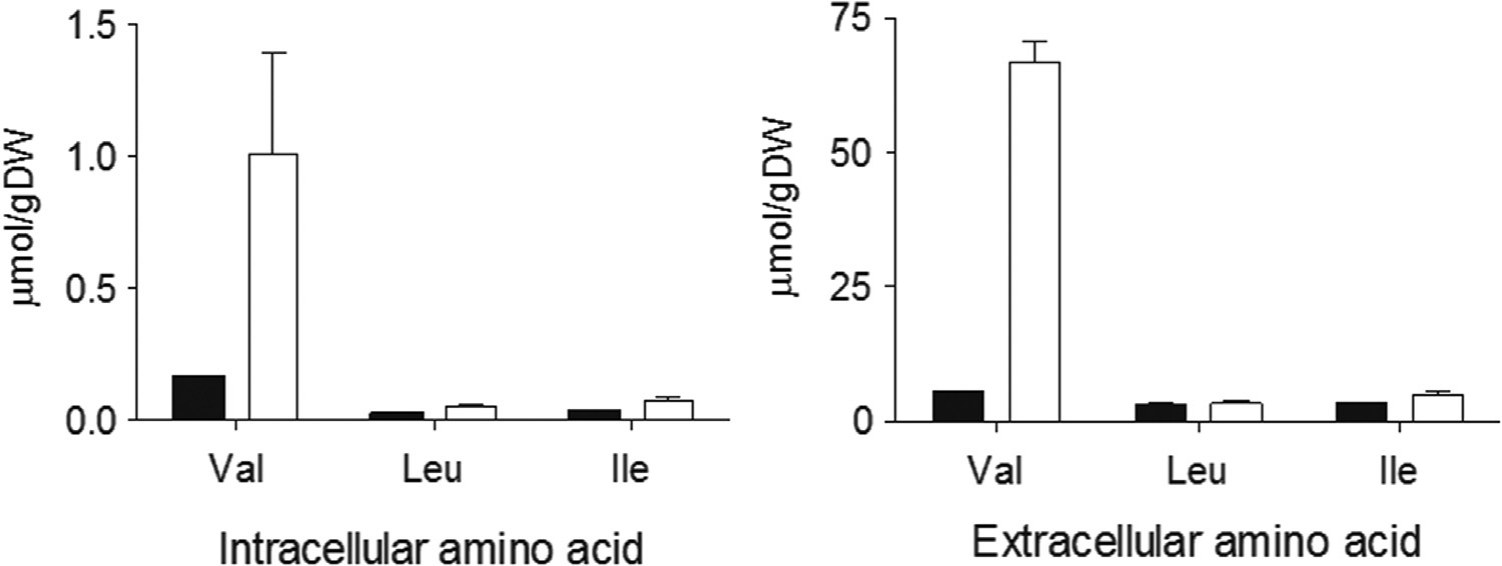
Intracellular and extracellular branched-chain amino acid pools in *S. cerevisiae* IMZ500 (Δ*pdc1,5,6 MTH1ΔT* p426GPD) (black bars) and IME305 (Δ*pdc1,5,6 Δilv2 MTH1ΔT*^*co*^*ilvBCDN* p426GPD) (white bars) expressed in µmol/gDW. Both strains were grown in SMG medium and samples taken over the course of exponential phase for analysis. Data are presented as averages and standard deviations of duplicate experiments.

To investigate whether expression of the cytosolic pathway led to branched-chain amino acid accumulation, strain IME305 and the Pdc^−^ reference strain IMZ500 (Δ*pdc1,5,6 MTH1ΔT* p426GPD) were grown in shake flask cultures on SMG medium, followed by analysis of intracellular and extracellular amino acid concentrations. In the reference strain IMZ500, concentrations of valine, leucine and isoleucine for IMZ500 were lower than intracellular branched chain amino acid concentrations measured precedently in a Pdc^+^ *S. cerevisiae* strain (Luttik et al. 2008) (Fig. 4). Significantly higher intra- and extracellular concentrations of valine, leucine and isoleucine were observed in cultures of strain IME305. In particular, intra- and extracellular valine concentrations were 6-fold and 12-fold higher, respectively, than in cultures of the reference strain IMZ500. These observations further confirmed the functionality of the engineered cytosolic pathway and, in particular, the successful bypassing of regulatory mechanisms that prevent valine accumulation in wild-type *S. cerevisiae* (Elisakova et al., 2005, Ljungdahl and Daignan-Fornier, 2012).

### 3.3 Physiological characterization of an engineered isobutanol pathway in *S. cerevisiae*

To complete the catabolic isobutanol pathway, a codon-optimized version of the *L. lactis* 2-oxo acid decarboxylase gene kdcA (Smit et al., 2005), which yields an active KIV decarboxylase upon expression in *S. cerevisiae* (Milne et al., 2015), was expressed from the episomal plasmid pUDE321 in IMX708. The resulting strain IME307 (Δ*pdc1,5,6 Δilv2 MTH1ΔT* ^*co*^*ilvN*^*M*^*13*^^ ^*co*^*ilvB* ^*co*^*ilvC*^*6E6 co*^*ilvD* ^*co*^*kdcA*) was then compared with strain IME305 and the Pdc^-^ reference strain IMZ500 in aerobic shake flask cultures on SMG medium.

Introduction of the heterologous pathway, either with or without *kdcA*, resulted in a 2.7 fold decrease of the specific growth rate relative to that of strain IMZ500 (Table 4). Presence of the heterologous pathway resulted in the formation of low quantities of isobutyrate as pictured by the calculated yield (Table 4). Although this result was far from the theoretical yield, it was however in full agreement with yields obtained in previous isobutanol engineering attempts in *S. cerevisiae* (Chen et al., 2011, Ida et al., 2015, Kondo et al., 2012, Matsuda et al., 2013, Park et al., 2014). Under aerobic conditions, *S. cerevisiae* preferably oxidizes isobutyraldehyde to isobutyrate (Hazelwood et al., 2008), which therefore can be taken as a proxy for isobutanol in these experiments. Consistent with an earlier report (van Maris et al., 2004a) the Pdc^−^ strain IMZ500 converted a large fraction of the consumed glucose to pyruvate (0.289±0.071 mol/mol glucose). Conversely, only trace amounts of pyruvate were detected extracellularly in cultures of strains IME305 and IME307 (<0.02 mol/mol glucose). Instead, these strains, which express the heterologous valine pathway, produced substantial concentrations of metabolites derived from the branched-chain amino acid pathway. In particular, they produced high extracellular concentrations of the pathway intermediate dihydroxyisovalerate (DHIV) and, in strain IME305, of KIV (Table 4). Additionally, diacetyl (derived from spontaneous oxidative decarboxylation of acetolactate (Suomalainen and Ronkainen, 1968)) and acetoin (produced from diacetyl by a diacetyl reductase (Ehsani et al., 2009)) were detected extracellularly (Table 4).

**Table 4:** Maximum specific growth rates, final optical density at 660 nm (OD660) and metabolite yields of aerobic shake flask cultures of *S. cerevisiae* strains IMZ500 (Δ*pdc1,5,6 MTH1ΔT* p426GPD), IME305 (Δ*pdc1,5,6 Δilv2 MTH1ΔT*^*co*^*ilvBCDN* p426GPD) and IME307 (Δ*pdc1,5,6 Δilv2*^MTH1ΔT *co*^*ilvBCDN*^*co*^*kdcA*). Cells were grown in SMG medium and samples taken for analysis over the course of the exponential phase. Data are presented as averages and mean deviation of duplicate experiments. *Sum total of extracellular diacetyl and acetolactate, BD: Below detection limit of analytical methods, DHIV: 2,3-dihydroxy-isovalerate, KIV: α-keto-isovalerate.

	IMZ500	IME305	IME307
	(*pdc* minus control)	(p426GPD)	(*kdcA*)
*µ*_max_ (h^−1^)	0.094±0.015	0.035±0.001	0.034±0.00
Final OD 660	4.07±0.31	4.84±0.27	2.78±0.11
Pyruvate (mol/mol glucose)	0.29±0.071	0.017±0.000	0.005±0.002
Diacetyl* (mol/mol glucose)	7.1.10^−^^5^±1.5.10^−^^5^	0.055±0.007	0.031±0.012
Acetoin (mol/mol glucose)	BD	0.18±0,00	0.09±0.02
DHIV (mol/mol glucose)	BD	0.31±0.00	0.19±0.07
KIV (mol/mol glucose)	BD	0.15±0.01	BD
Isobutyrate (mol/mol glucose)	BD	0.02±0.00	0.05±0.00
Isobutanol (mol/mol glucose)	BD	BD	BD

Quantitative comparison of the strains in shake flasks was complicated by accumulation of organic acids (e.g. pyruvate, DHIV, KIV and isobutyrate), which led to acidification and cessation of growth before glucose was fully consumed. Therefore, aerobic, pH-controlled bioreactor cultures were performed with strains IME307 (Δ*pdc156 Δilv2 MTH1ΔT* ^*co*^*ilvN*^*M13 co*^*ilvB* ^*co*^*ilvC*^*6E6 co*^*ilvD* ^*co*^*kdcA*) and IMZ500 (Δ*pdc156 MTH1ΔT* p426GPD) to quantify metabolic fluxes through the cytosolic isobutanol pathway and towards the observed by-products. In the bioreactor cultures, glucose was completely consumed by both strains. As well as a decreased growth rate, IME307 displayed a substantially lower biomass yield (0.136±0.021 g/g glucose) than IMZ500 (0.422±0.012 g/g glucose), and a concomitant decrease in qCO_2_ (0.089±0.019 g/g biomass/h for IME307 versus 0.165±0.012 g/g biomass/h for IMZ500) (Table 5). In general, metabolite profiles of the two strains in bioreactors strongly resembled those observed in shake flasks, with the exception of the production of some α-ketoisovalerate and a higher acetoin yield in strain IME307.

**Table 5:** Physiology and metabolite production of *S. cerevisiae* strains IMZ500 (Δ*pdc1,5,6 MTH1ΔT* p426GPD) and IME307 (Δ*pdc1,5,6 Δilv2 MTH1ΔT*^*co*^*ilvBCDN*^*co*^*kdcA*) in aerobic batch cultures on SMG medium maintained at pH 5.0. Data are presented as average and mean deviation of duplicate experiments. *Sum total of extracellular diacetyl and acetolactate, BD: Below detection limit of analytical methods, DHIV: 2,3-dihydroxy-isovalerate, KIV: α-keto-isovalerate.

	IMZ500	IME307
Growth rate (h^−1^)	0.115±0.010	0.020±0.001
*Y*_X/S_ (g/g glucose)	0.422±0.012	0.136±0.021
qGlucose (g/g biomass/h)	−0.273±0.030	−0.152±0.031
qCO_2_ (g/g biomass/h)	0.165±0.012	0.089±0.019
Pyruvate yield (mol/mol glucose)	0.330±0.001	0.001±0.000
Ethanol yield (mol/mol glucose)	BD	BD
Diacetyl yield^*^ (mol/mol glucose)	0.001±0.000	0.040±0.002
Acetoin yield (mol/mol glucose)	BD	0.053±0.002
DHIV yield (mol/mol glucose)	BD	0.201±0.010
KIV yield (mol/mol glucose)	BD	0.033±0.001
Isobutyrate yield (mol/mol glucose)	BD	0.021±0.004
Isobutanol yield (mol/mol glucose)	BD	BD
Carbon recovery (%)	103.4±4.7	103.2±2.8

### 3.4 Distribution of carbon flux in micro-aerobic cultures

Oxygen availability not only affects the conversion of isobutyraldehyde to either isobutanol or isobutyrate (Hazelwood et al*.*, 2008), but also influences ATP generation and NADH oxidation via respiration. Although the isobutanol pathway used in this study was designed to function as a catabolic pathway, strain IME307, which expresses the complete pathway, did not show growth on glucose in anaerobic cultures. Growth remained absent when cultures were incubated for several weeks in an attempt to select for spontaneous mutants in which the capacity and/or other characteristics of the engineered pathway had improved sufficiently to sustain anaerobic growth. Therefore, further analysis of the pathway was performed in micro-aerobic high-cell-density cultures, using biomass from aerobic, pH-controlled bioreactor cultures. The absence of growth in these micro-aerobic cultures facilitated a stoichiometric analysis of flux distribution. Micro-aerobically, isobutanol production was observed in IME307, but at a very low yield (0.018±0.003 mol/mol glucose) (Table 6). However, isobutyrate was still produced at higher yields (0.065±0.005 mol/mol glucose). Acetoin was not detected in the micro-aerobic cultures. Instead, 2,3-butanediol, the product of acetoin reduction, was produced at very high yields (0.65±0.07 mol/mol glucose), indicating that the micro-aerobic conditions favoured reduction of acetoin to 2,3-butanediol. Glycerol production was observed in both strains. In strain IMZ500 (Δ*pdc1,5,6 MTH1ΔT* p426GPD), glycerol production can be attributed to the need to re-oxidize the NADH formed as a result of pyruvate accumulation. In strain IME307 (Δ*pdc1,5,6 Δilv2 MTH1ΔT* ^*co*^*ilvN*^*M*^*13*^^ ^*co*^*ilvB* ^*co*^*ilvC*^*6E6 co*^*ilvD* ^*co*^*kdcA*), which produced much lower concentrations of pyruvate, the NADH required for glycerol production was likely derived from the formation of oxidised products DHIV, isobutyrate and CO_2_. In strain IME307, a low but significant production of ethanol was observed, consistent with the low affinity of KdcA towards pyruvate (Milne et al., 2015).

**Table 6:** Metabolite production from glucose bio-conversion in micro-aerobic cultures of IMZ500 (Δ*pdc1,5,6 MTH1ΔT* p426GPD) and IME307 (Δ*pdc1,5,6 Δilv2 MTH1ΔT*^*co*^*ilvBCDN*^*co*^*kdcA*). Cells were first grown in SMG medium in aerobic, pH-controlled bioreactors, then washed with water and resuspended to a final cell density of ~12 g/L in SMG medium supplemented with Tween-80 (420 mg/L) and ergosterol (10 mg/L) with the initial pH set to 6.0. Cells were then incubated micro-aerobically at 30 °C and metabolite concentrations were measured during linear glucose consumption. Data are presented as average and mean deviation of duplicate experiments. *Sum total of extracellular diacetyl and acetolactate, BD: Below detection limit of analytical methods, NA: Not applicable, ND: Not determined, DHIV: 2,3-dihydroxy-isovalerate, KIV: α-keto-isovalerate.

	IMZ500		IME307	
	Rate	Yield	Rate	Yield
	(µmol/g biomass/h)	(mol/mol glucose)	(µmol/g biomass/h)	(mol/mol glucose)
Glucose	−155.74±11.00	NA	−25.95±4.57	NA
Pyruvate	116.66±0.94	0.770±0.101	1.08±0.28	0.041±0.013
Ethanol	BD	BD	3.37±0.75	0.131±0.039
Glycerol	123.42±25.36	0.76±0.00	11.73±3.41	0.51±0.00
Acetoin	BD	BD	BD	BD
2,3-butanediol	BD	BD	13.66±1.70	0.649±0.067
DHIV	BD	BD	1.71±0.30	0.070±0.000
KIV	BD	BD	BD	ND
Isobutyrate	BD	BD	1.40±0.12	0.065±0.005
Isobutanol	BD	BD	0.41±0.09	0.018±0.003

**Fig. 5:**
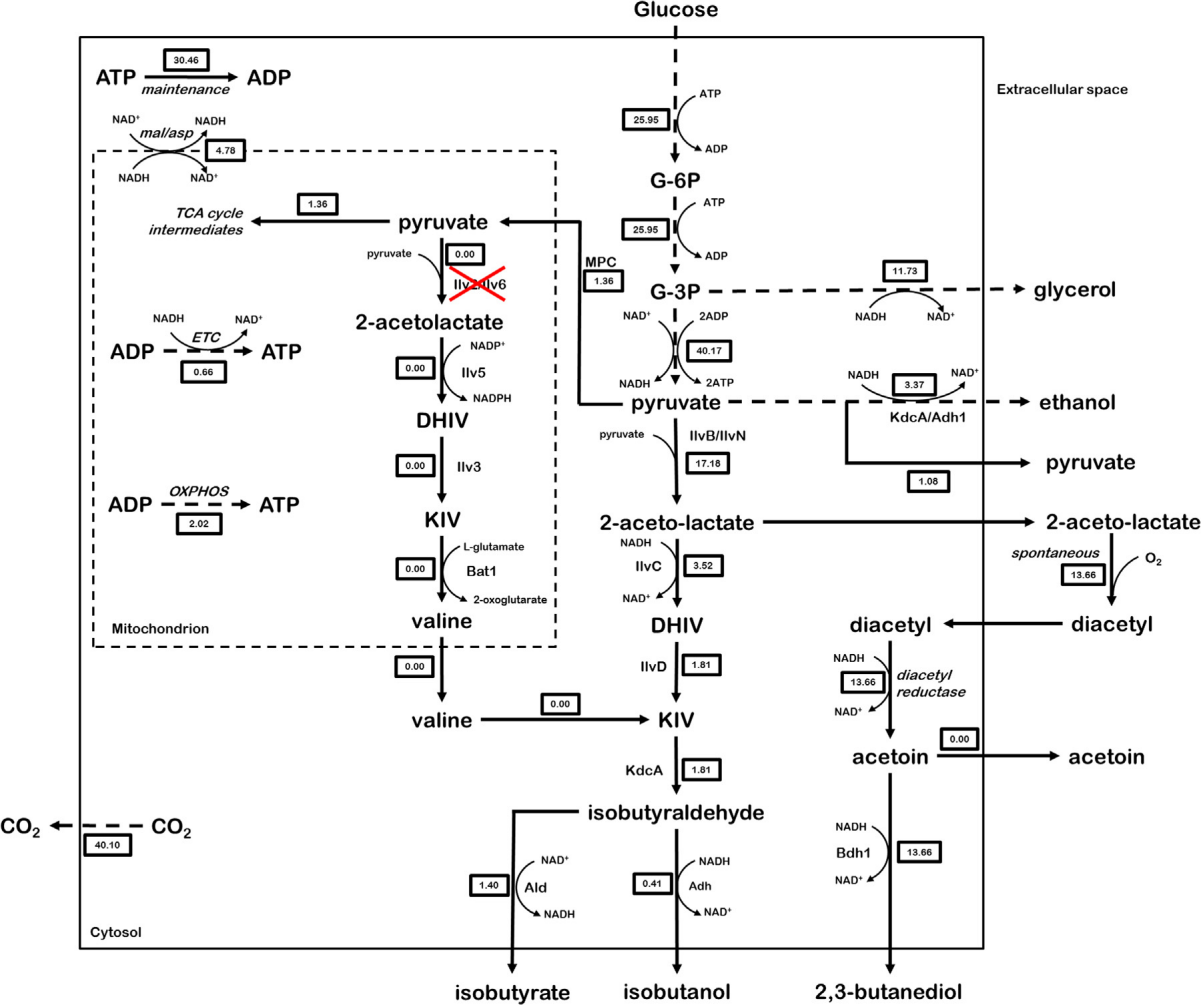
Flux distribution maps for *S. cerevisiae* IME307 (Δ*pdc1,5,6 Δilv2 MTH1ΔT*^*co*^*ilvBCDN*^*co*^*kdcA*) grown in micro-aerobic cultures (see Table 6), calculated using CellNetAnalyzer. Dashed arrows represent multiple enzyme-catalysed reactions. Numbered boxes represent the modelled metabolic flux through each reaction (expressed in µmol/g biomass/h). Mal/asp: malate/aspartate shuttle, ETC: Electron transport chain, OXPHOS: Oxidative phosphorylation, G-6P: glucose-6-phosphate, G-3P: glyceraldehyde-3-phosphate, DHIV: 2,3-dihydroxyisovalerate, KIV: α-ketoisovalerate.

A stoichiometric metabolic model was set up to visualize the distribution of glucose carbon over the intracellular pathways leading to the surprisingly large set of (by-)products observed in these experiments. Using the biomass-specific rates of extracellular product formation as input (Table 6), the model allowed for the construction of detailed intracellular flux maps (Fig. 5). These flux maps indicated a low activity of the TCA cycle, respiration and oxidative phosphorylation in the micro-aerobic cultures. Approximately 45% of the total carbon flux was diverted to glycerol in order to maintain a redox-cofactor balance. At the acetolactate branch point, a significant fraction (80%) of the remaining carbon flux was diverted away from the isobutanol pathway and into the 2,3-butanediol pathway. The model enabled an estimation of the specific rate of ATP synthesis from glycolysis and oxidative phosphorylation of 0.03 mMol/g biomass/h. With an estimated ATP requirement for cellular maintenance of anaerobic *S. cerevisiae* cultures of ca. 1 mMol/g biomass/h (Boender *et al.*, 2009), the *in vivo* rate of ATP production from the engineered isobutanol pathway was clearly too low to sustain anaerobic growth on glucose.

## 4 Discussion

Expression in *S. cerevisiae* of a set of heterologous enzymes that, theoretically, should be able to form a catabolic isobutanol pathway, resulted in low isobutanol yields (0.018±0.003 mol/mol glucose). The specific rate of isobutanol production by the engineered strain was too low to meet the cellular maintenance energy requirement and, consequently, did not support anaerobic growth. These results were similar to those obtained in previous academic studies on metabolic engineering of *S. cerevisiae*. A systematic mass balancing approach revealed massive accumulation of pathway intermediates and related metabolites.

The observation that, in micro-aerobic cultures, the yield of isobutyrate (0.065±0.005 mol/mol glucose) exceeded that of isobutanol is consistent with a previously reported limitation at the isobutyraldehyde branch-point (Park et al., 2014). Production of diacetyl, acetoin and 2,3-butanediol, the latter reaching a considerably high yield of 0.649±0.067 mol/mol glucose in aerobic cultures, identified a previously unreported ‘overflow’ at the level of acetolactate. This result indicates that the feedback-insensitive bacterial acetolactate synthase (^*co*^*ilvN*^*M*^*13*^^*,* ^*co*^*ilvB*) was fully functional in the engineered strain, but that a significant limitation occurred downstream of acetolactate. Analysis of metabolic fluxes in micro-aerobic cultures indicated that production of KIV was significantly slower than that of DHIV. In the engineered strain, conversion of DHIV to KIV was catalysed by the dihydroxyacid dehydratase IlvD. Prokaryotic and eukaryotic dihydroxyacid dehydratases contain iron–sulphur (4Fe–4S) clusters and require iron–sulphur cluster biogenesis and assembly mechanisms for *in vivo* activity (Flint et al., 1993, Lill, 2009, Muhlenhoff et al., 2011, Rouault and Tong, 2005). In *S. cerevisiae*, iron–sulphur cluster biogenesis and assembly into mature proteins occurs predominantly in the mitochondrial matrix (Schilke et al., 1999), the location of the native yeast dihydroxyacid dehydratase Ilv3. Iron–sulphur cluster assembly can also occur in the yeast cytosol (Carlsen et al., 2013, Kozak et al., 2014a, Waks and Silver, 2009), but has a much lower capacity than the mitochondrial system (Brat et al., 2012, Sharma et al., 2010). Limitation of the *in vivo* activity of IlvD by biogenesis and assembly of its 4Fe-4S cluster is entirely consistent with low rate of KIV production observed in strain IME307. Moreover, it has also proven to be difficult to express other pathways that rely on cytosolic iron–sulphur cluster assembly in the yeast cytosol (Benisch and Boles, 2014, Carlsen et al., 2013).

However, we cannot exclude as well other secondary bottlenecks in the pathway that might require special attention in the future. Under the fermentation conditions used in the study, (oxygen limited to microaerobic), the main alcohol dehydrogenase expressed should be *ADH1* (Knijnenburg et al., 2009). However, Adh1 has a non-optimal conversion kinetics of isobutyraldehyde. Overexpresion of the native Adh2 alcohol dehydrogenase which exhibits a five-fold higher rate than Adh1 for isobutyraldehyde has been shown to have a positive impact on isobutanol formation (Brat et al., 2012). Although significant the isobutanol improvement resulting from the overexpression of Adh2, or other alcohol dehydrogenases e.g. Adh6, Adh7, AdhA) (Brat et al., 2012, Kondo et al., 2012, Avalos et al., 2013, Matsuda et al., 2013) remained limited suggesting that this step while contributing to the overall flux did not represent the main controlling step of the pathway.

The extracellular accumulation of acetolactate, DHIV and KIV indicate the presence of export mechanisms for these pathway intermediates in the yeast plasma membrane. Consistent with the multi-genic nature of the transport of other carboxylic acids in *S. cerevisiae* (de Kok et al., 2012), screening of single deletion mutants failed to identify a unique acetolactate transporter (Dundon et al., 2011b). Even in the absence of kinetic limitations in the isobutanol pathway, export of its intermediates might interfere with efficient performance in *S. cerevisiae*. In *S. cerevisiae*, export of carboxylic acids remains a poorly understood subject, as exemplified by the fact that even export of simple organic acids such as acetic acid and lactic acid remain incompletely understood (Casal et al., 2008, Paiva et al., 2004, van Maris et al., 2004b). Identification and inactivation of transporters for pathway intermediates may therefore be relevant for further development of isobutanol-producing yeast strains.

A series of patent applications related to cytosolic iron–sulphur cluster availability (Dundon et al*.*, 2011a), reducing extracellular accumulation of metabolites (Buelter et al., 2012, Dundon et al., 2011b), and improving the enzyme kinetics of the isobutanol pathway (Li et al., 2010, Liao et al., 2013, Porter-Scheinman et al., 2014) indicates that industrial researchers have, in all likelihood, already made substantial progress in addressing several of the issues indicated above. This notwithstanding, the present study helps to interpret the outcome of earlier academic studies and underlines the importance of a systematic, mass-balancing based approach in metabolic engineering studies.

Expression of ^*co*^*ilvB,* ^*co*^*ilvN*^*M*^*13*^^ (*C. glutamicum*), ^*co*^*ilvC*^*6E6*^ (*E. coli*)*,* and ^*co*^*ilvD* (*L. lactis*) in *S. cerevisiae* strains harbouring individual deletions in the native valine biosynthesis pathway restored branched-chain amino acid prototrophy. Although originally designed to merely test the *in vivo* functionality of the heterologous genes used to assemble the isobutanol pathway, these experiments yielded new insights into branched-chain amino acid metabolism in *S. cerevisiae*. Firstly, cytosolic expression of the complete pathway led to a significant increase of intra- and extracellular valine concentrations. To our knowledge, this is the first demonstration that valine production in *S. cerevisiae* can be increased by bypassing the regulatory mechanisms of its native biosynthesis pathway. Secondly, the complementation of branched-chain amino acid auxotrophs indicates that either (i) the native gene deletion is complemented by its heterologous counter-part, implying that intermediates of the branched-chain amino acid biosynthesis pathway(s) can cross the mitochondrial membrane, and/or (ii) the complete cytosolic pathway is active and able to cytosolically produce valine, leucine and isoleucine. The engineered strains described in this study offer a unique experimental platform for introduction of additional mutations to explore trafficking of precursors, intermediates, and products of the branched-chain amino acid biosynthesis pathway between yeast cytosol and mitochondria.
