# Supplementary material for: Excessive by-product formation: A key contributor to low isobutanol yields of engineered Saccharomyces cerevisiae strains
Source: Metab Eng Commun. 2016 Jan 20;3:39–51. doi: 10.1016/j.meteno.2016.01.002 (PMC5678825; doi:10.1016/j.meteno.2016.01.002)
Supplement: Supplementary file 1 — Supplementary material [file mmc1.docx]

**Supplementary material 1.** Metabolic modelling of “wild-type” (A) and catabolic (B) isobutanol producing pathways in *S. cerevisiae* graphically represented using Omix.


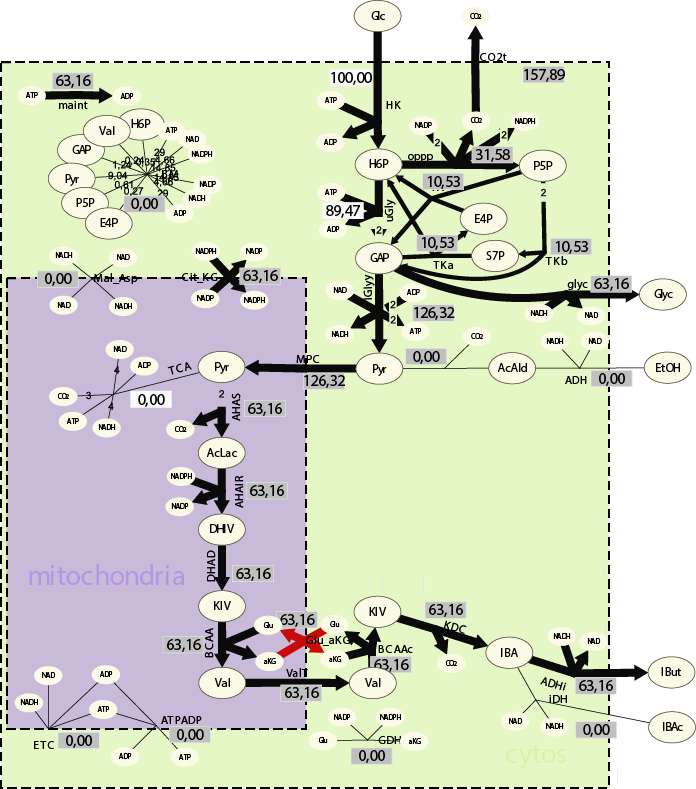

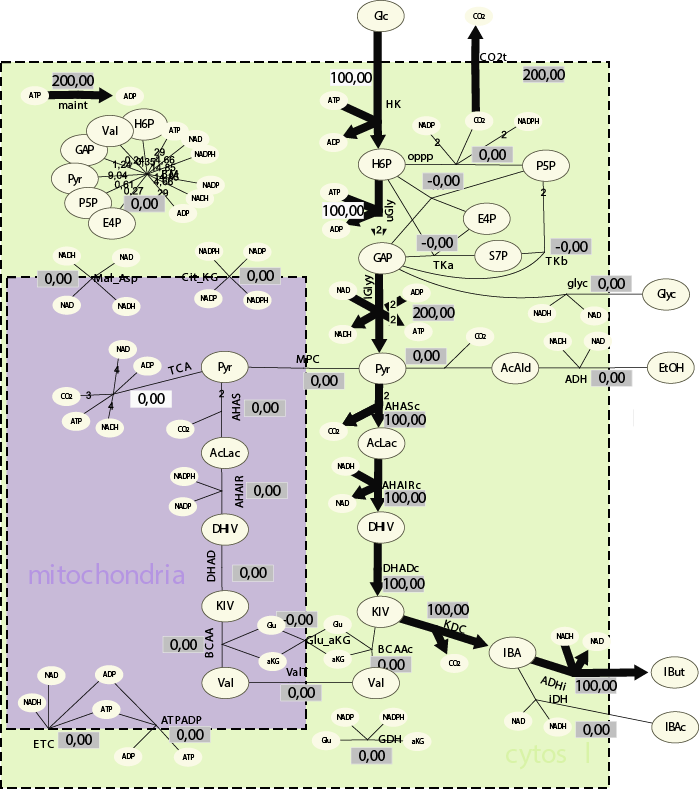


**Supplementary material 2.** Metabolic flux analysis (expressed in µmol/g biomass/h) using the extracellular metabolite concentrations obtained from IME307 (Δ*pdc1* Δ*pdc5* Δ*pdc6 Δilv2* MTH1ΔT *ilvBCDN* pUDE321) in the micro-aerobic glucose bio-conversion experiment analysed using CellNetAnalyzer and graphically represented using Omix.


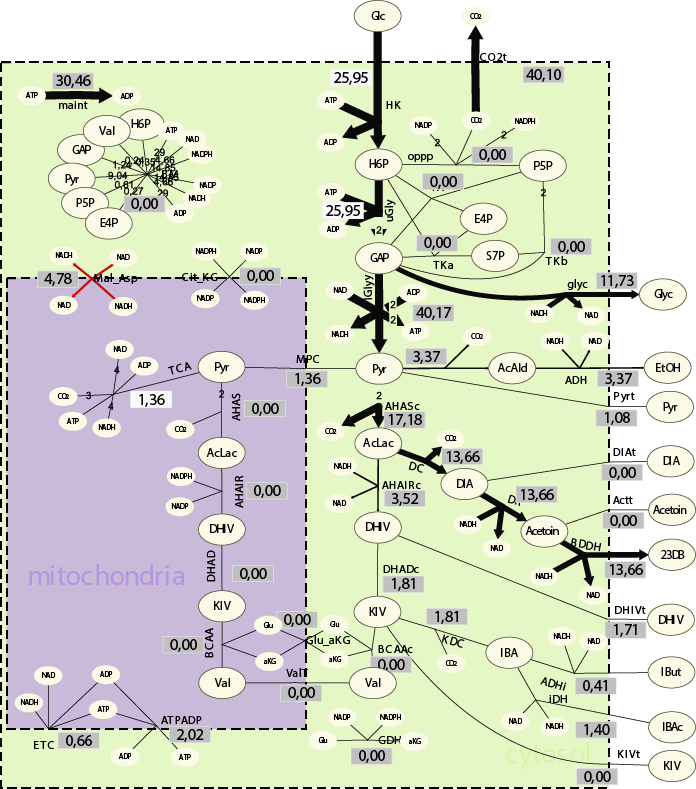


**Supplementary material 3.** List of reactions used in metabolic models.

Reaction 1: uGly: H6P + ATP ==> 2 GAP + ADP

Reaction 2: HK: ATP + Glc_e ==> H6P + ADP

Reaction 3: TCA: Pyr_m + 4 NAD_m + ADP_m ==> 3 CO2 + 4 NADH_m + ATP_m

Reaction 4: lGlyy: GAP + NAD + 2 ADP ==> Pyr + NADH + 2 ATP

Reaction 5: AHAS: 2 Pyr_m ==> CO2 + AcLac_m

Reaction 6: AHAIR: AcLac_m + NADPH_m ==> DHIV_m + NADP_m

Reaction 7: DHAD: DHIV_m ==> KIV_m

Reaction 8: BCAA: KIV_m + Glu_m <==> Val_m + aKG_m

Reaction 9: MPC: Pyr <==> Pyr_m

Reaction 10: oppp: H6P + 2 NADP ==> CO2 + 2 NADPH + P5P

Reaction 11: TA: P5P + E4P <==> GAP + H6P

Reaction 12: TKa: GAP + S7P <==> H6P + E4P

Reaction 13: TKb: 2 P5P <==> GAP + S7P

Reaction 14: PDC: Pyr ==> CO2 + AcAld

Reaction 15: ADH: NADH + AcAld ==> NAD + EtOH_e

Reaction 16: AHASc: 2 Pyr ==> CO2 + AcLac

Reaction 17: AHAIRc: AcLac + NADH ==> DHIV + NAD

Reaction 18: DHADc: DHIV ==> KIV

Reaction 19: KDC: KIV ==> CO2 + IBA

Reaction 20: ADHi: NADH + IBA ==> NAD + IBut_e

Reaction 21: ValT: Val_m ==> Val

Reaction 22: BCAAc: Val + aKG <==> KIV + Glu

Reaction 23: Cit_KG: NADPH + NADP_m <==> NADPH_m + NADP

Reaction 24: ETC: NADH_m + ADP_m ==> NAD_m + ATP_m

Reaction 25: ATPADP: ATP_m + ADP <==> ATP + ADP_m

Reaction 26: DC: AcLac + 0.5O_2_ ==> CO2 + DIA

Reaction 27: DR: NADH + DIA ==> NAD + Acetoin

Reaction 28: BDDH: NADH + Acetoin ==> NAD + 23DB_e

Reaction 29: DIAt: DIA ==> DIA_e

Reaction 30: Actt: Acetoin ==> Acetoin_e

Reaction 31: Pyrt: Pyr ==> Pyr_e

Reaction 32: DHIVt: DHIV ==> DHIV_e

Reaction 33: KIVt: KIV ==> KIV_e

Reaction 34: CO2t: CO2 ==> CO2_e

Reaction 35: Glu_aKG: Glu_m + aKG <==> Glu + aKG_m

Reaction 36: maint: ATP ==> ADP

Reaction 37: Mal_Asp: NADH + NAD_m <==> NADH_m + NAD

Reaction 38: BM: 1.24 GAP + 0.35 H6P + 9.04 Pyr + 0.24 Val + 14.85 NADPH + 4.66 NAD + 29 ATP + 0.61 P5P + 0.27 E4P ==> 14.85 NADP + 4.66 NADH + 29 ADP

Reaction 39: GDH: aKG + NADPH ==> Glu + NADP

Reaction 40: glyc: GAP + NADH ==> Glyc_e + NAD

Reaction 41: iDH: NAD + IBA ==> NADH + IBAc

**Supplementary material 4.** DNA sequences of codon optimized custom synthesized DNA cassettes.

*TPI1_P_-*co*ilvB-ADH1_t_*

GATCTACGTATGGTCATTTCTTCTTCAGATTCCCTCATGGAGAAAGTGCGGCAGATGTATATGACAGAGTCGCCAGTTTCCAAGAGACTTTATTCAGGCACTTCCATGATAGGCAAGAGAGAAGACCCAGAGATGTTGTTGTCCTAGTTACACATGGTATTTATTCCAGAGTATTCCTGATGAAATGGTTTAGATGGACATACGAAGAGTTTGAATCGTTTACCAATGTTCCTAACGGGAGCGTAATGGTGATGGAACTGGACGAATCCATCAATAGATACGTCCTGAGGACCGTGCTACCCAAATGGACTGATTGTGAGGGAGACCTAACTACATAGTGTTTAAAGATTACGGATATTTAACTTACTTAGAATAATGCCATTTTTTTGAGTTATAATAATCCTACGTTAGTGTGAGCGGGATTTAAACTGTGAGGACCTTAATACATTCAGACACTTCTGCGGTATCACCCTACTTATTCCCTTCGAGATTATATCTAGGAACCCATCAGGTTGGTGGAAGATTACCCGTTCTAAGACTTTTCAGCTTCCTCTATTGATGTTACACCTGGACACCCCTTTTCTGGCATCCAGTTTTTAATCTTCAGTGGCATGTGAGATTCTCCGAAATTAATTAAAGCAATCACACAATTCTCTCGGATACCACCTCGGTTGAAACTGACAGGTGGTTTGTTACGCATGCTAATGCAAAGGAGCCTATATACCTTTGGCTCGGCTGCTGTAACAGGGAATATAAAGGGCAGCATAATTTAGGAGTTTAGTGAACTTGCAACATTTACTATTTTCCCTTCTTACGTAAATATTTTTCTTTTTAATTCTAAATCAATCTTTTTCAATTTTTTGTTTGTATTCTTTTCTTGCTTAAATCTATAACTACAAAAAACACATATGAACGTTGCTGCTTCTCAACAACCAACTCCAGCTACTGTTGCTTCTAGAGGTAGATCTGCTGCTCCAGAAAGAATGACTGGTGCTAAGGCTATCGTTAGATCTTTGGAAGAATTGAACGCTGACATCGTTTTCGGTATCCCAGGTGGTGCTGTTTTGCCAGTTTACGACCCATTGTACTCTTCTACTAAGGTTAGACACGTTTTGGTTAGACACGAACAAGGTGCTGGTCACGCTGCTACTGGTTACGCTCAAGTTACTGGTAGAGTTGGTGTTTGTATCGCTACTTCTGGTCCAGGTGCTACTAACTTGGTTACTCCAATCGCTGACGCTAACTTGGACTCTGTTCCAATGGTTGCTATCACTGGTCAAGTTGGTTCTGGTTTGTTGGGTACTGACGCTTTCCAAGAAGCTGACATCAGAGGTATCACTATGCCAGTTACTAAGCACAACTTCATGGTTACTAACCCAAACGACATCCCACAAGCTTTGGCTGAAGCTTTCCACTTGGCTATCACTGGTAGACCAGGTCCAGTTTTGGTTGACATCCCAAAGGACGTTCAAAACGCTGAATTGGACTTCGTTTGGCCACCAAAGATCGACTTGCCAGGTTACAGACCAGTTTCTACTCCACACGCTAGACAAATCGAACAAGCTGTTAAGTTGATCGGTGAAGCTAAGAAGCCAGTTTTGTACGTTGGTGGTGGTGTTATCAAGGCTGACGCTCACGAAGAATTGAGAGCTTTCGCTGAATACACTGGTATCCCAGTTGTTACTACTTTGATGGCTTTGGGTACTTTCCCAGAATCTCACGAATTGCACATGGGTATGCCAGGTATGCACGGTACTGTTTCTGCTGTTGGTGCTTTGCAAAGATCTGACTTGTTGATCGCTATCGGTTCTAGATTCGACGACAGAGTTACTGGTGACGTTGACACTTTCGCTCCAGACGCTAAGATCATCCACGCTGACATCGACCCAGCTGAAATCGGTAAGATCAAGCAAGTTGAAGTTCCAATCGTTGGTGACGCTAGAGAAGTTTTGGCTAGATTGTTGGAAACTACTAAGGCTTCTAAGGCTGAAACTGAAGACATCTCTGAATGGGTTGACTACTTGAAGGGTTTGAAGGCTAGATTCCCAAGAGGTTACGACGAACAACCAGGTGACTTGTTGGCTCCACAATTCGTTATCGAAACTTTGTCTAAGGAAGTTGGTCCAGACGCTATCTACTGTGCTGGTGTTGGTCAACACCAAATGTGGGCTGCTCAATTCGTTGACTTCGAAAAGCCAAGAACTTGGTTGAACTCTGGTGGTTTGGGTACTATGGGTTACGCTGTTCCAGCTGCTTTGGGTGCTAAGGCTGGTGCTCCAGACAAGGAAGTTTGGGCTATCGACGGTGACGGTTGTTTCCAAATGACTAACCAAGAATTGACTACTGCTGCTGTTGAAGGTTTCCCAATCAAGATCGCTTTGATCAACAACGGTAACTTGGGTATGGTTAGACAATGGCAAACTTTGTTCTACGAAGGTAGATACTCTAACACTAAGTTGAGAAACCAAGGTGAATACATGCCAGACTTCGTTACTTTGTCTGAAGGTTTGGGTTGTGTTGCTATCAGAGTTACTAAGGCTGAAGAAGTTTTGCCAGCTATCCAAAAGGCTAGAGAAATCAACGACAGACCAGTTGTTATCGACTTCATCGTTGGTGAAGACGCTCAAGTTTGGCCAATGGTTTCTGCTGGTTCTTCTAACTCTGACATCCAATACGCTTTGGGTTTGAGACCATTCTTCGACGGTGACGAATCTGCTGCTGAAGACCCAGCTGACATCCACGAAGCTGTTTCTGACATCGACGCTGCTGTTGAATCTACTGAAGCTTAAGCGAATTTCTTATGATTTATGATTTTTATTA

TTAAATAAGTTATAAAAAAAATAAGTGTATACAAATTTTAAAGTGACTCTTAGGTTTTAAAACGAAAATTCTTATTCTTGAGTAACTCTTTCCTGTAGGTCAGGTTGCTTTCTCAGGTATAGCATGAGGTCGCTCTTATTGACCACACCTCTACCGGCATGCCGAGCAAATGCCTGCAAATCGCTCCCCATTTCACCCAATTGTAGATATGCTAACTCCAGCAATGAGTTGATGAATCTCGGTGTGTATTTTATGTCCTCAGAGGACAACACCTGTTGTAATCGTTCTTCCACAC

*ADH1_P_-coilvC^6E6^-PYK1_t_*

TAAAACAAGAAGAGGGTTGACTACATCACGATGAGGGGGATCGAAGAAATGATGGTAAATGAAATAGGAAATCAAGGAGCATGAAGGCAAAAGACAAATATAAGGGTCGAACGAAAAATAAAGTGAAAAGTGTTGATATGATGTATTTGGCTTTGCGGCGCCGAAAAAACGAGTTTACGCAATTGCACAATCATGCTGACTCTGTGGCGGACCCGCGCTCTTGCCGGCCCGGCGATAACGCTGGGCGTGAGGCTGTGCCCGGCGGAGTTTTTTGCGCCTGCATTTTCCAAGGTTTACCCTGCGCTAAGGGGCGAGATTGGAGAAGCAATAAGAATGCCGGTTGGGGTTGCGATGATGACGACCACGACAACTGGTGTCATTATTTAAGTTGCCGAAAGAACCTGAGTGCATTTGCAACATGAGTATACTAGAAGAATGAGCCAAGACTTGCGAGACGCGAGTTTGCCGGTGGTGCGAACAATAGAGCGACCATGACCTTGAAGGTGAGACGCGCATAACCGCTAGAGTACTTTGAAGAGGAAACAGCAATAGGGTTGCTACCAGTATAAATAGACAGGTACATACAACACTGGAAATGGTTGTCTGTTTGAGTACGCTTTCAATTCATTTGGGTGTGCACTTTATTATGTTACAATATGGAAGGGAACTTTACACTTCTCCTATGCACATATATTAATTAAAGTCCAATGCTAGTAGAGAAGGGGGGTAACACCCCTCCGCGCTCTTTTCCGATTTTTTTCTAAACCGTGGAATATTTCGGATATCCTTTTGTTGTTTCCGGGTGTACAATATGGACTTCCTCTTTTCTGGCAACCAAACCCATACATCGGGATTCCTATAATACCTTCGTTGGTCTCCCTAACATGTAGGTGGCGGAGGGGAGATATACAATAGAACAGATACCAGACAAGACATAATGGGCTAAACAAGACTACACCAATTACACTGCCTCATTGATGGTGGTACATAACGAACTAATACTGTAGCCCTAGACTTGATAGCCATCATCATATCGAAGTTTCACTACCCTTTTTCCATTTGCCATCTATTGAAGTAATAATAGGCGCATGCAACTTCTTTTCTTTTTTTTTCTTTTCTCTCTCCCCCGTTGTTGTCTCACCATATCCGCAATGACAAAAAAATGATGGAAGACACTAAAGGAAAAAATTAACGACAAAGACAGCACCAACAGATGTCGTTGTTCCAGAGCTGATGAGGGGTATCTCGAAGCACACGAAACTTTTTCCTTCCTTCATTCACGCACACTACTCTCTAATGAGCAACGGTATACGGCCTTCCTTCCAGTTACTTGAATTTGAAATAAAAAAAAGTTTGCTGTCTTGCTATCAAGTATAAATAGACCTGCAATTATTAATCTTTTGTTTCCTCGTCATTGTTCTCGTTCCCTTTCTTCCTTGTTTCTTTTTCTGCACAATATTTCAAGCTATACCAAGCATACAATCAACTATCTCATATACAATGGCTAACTACTTCAACACTTTGAACTTGAGACAACAATTGGCTCAATTGGGTAAGTGTAGATTCATGGGTAGAGACGAATTCGCTGACGGTGCTTCTTACTTGCAAGGTAAGAAGGTTGTTATCGTTGGTTGTGGTGCTCAAGGTTTGAACCAAGGTTTGAACATGAGAGACTCTGGTTTGGACATCTCTTACGCTTTGAGAAAGGAATCTATCGCTGAAAAGGACGCTGACTGGAGAAAGGCTACTGAAAACGGTTTCAAGGTTGGTACTTACGAAGAATTGATCCCACAAGCTGACTTGGTTATCAACTTGACTCCAGACAAGGTTCACTCTGACGTTGTTAGAACTGTTCAACCATTGATGAAGGACGGTGCTGCTTTGGGTTACTCTCACGGTTTCAACATCGTTGAAGTTGGTGAACAAATCAGAAAGGACATCACTGTTGTTATGGTTGCTCCAAAGTGTCCAGGTACTGAAGTTAGAGAAGAATACAAGAGAGGTTTCGGTGTTCCAACTTTGATCGCTGTTCACCCAGAAAACGACCCAAAGGGTGAAGGTATGGCTATCGCTAAGGCTTGGGCTGCTGCTACTGGTGGTCACAGAGCTGGTGTTTTGGAATCTTCTTTCGTTGCTGAAGTTAAGTCTGACTTGATGGGTGAACAAACTATCTTGTGTGGTATGTTGCAAGCTGGTTCTTTGTTGTGTTTCGACAAGTTGGTTGAAGAAGGTACTGACCCAGCTTACGCTGAAAAGTTGATCCAATTCGGTTGGGAAACTATCACTGAAGCTTTGAAGCAAGGTGGTATCACTTTGATGATGGACAGATTGTCTAACCCAGCTAAGTTGAGAGCTTACGCTTTGTCTGAACAATTGAAGGAAATCATGGCTCCATTGTTCCAAAAGCACATGGACGACATCATCTCTGGTGAATTCTCTTCTGGTATGATGGCTGACTGGGCTAACGACGACAAGAAGTTGTTGACTTGGAGAGAAGAAACTGGTAAGACTGCTTTCGAAACTGCTCCACAATACGAAGGTAAGATCGGTGAACAAGAATACTTCGACAAGGGTGTTTTGATGATCGCTATGGTTAAGGCTGGTGTTGAATTGGCTTTCGAAACTATGGTTGACTCTGGTATCATCGAAGAATCTGCTTACTACGAATCTTTGCACGAATTGCCATTGATCGCTAACACTATCGCTAGAAAGAGATTGTACGAAATGAACGTTGTTATCTCTGACACTGCTGAATACGGTAACTACTTGTTCTCTTACGCTTGTGTTCCATTGTTGAAGCCATTCATGGCTGAATTGCAA

CCAGGTGACTTGGGTAAGGCTATCCCAGAAGGTGCTGTTGACAACGGTCAATTGAGAGACGTTAACGAAGCTATCAGATCTCACGCTATCGAACAAGTTGGTAAGAAGTTGAGAGGTTACATGACTGACATGAAGAGAATCGCTGTTGCTGGTTAAAAAAAGAATCATGATTGAATGAAGATATTATTTTTTTGAATTATATTTTTTAAATTTTATATAAAGACATGGTTTTTCTTTTCAACTCAAATAAAGATTTATAAGTTACTTAAATAACATACATTTTATAAGGTATTCTATAAAAAGAGTATTATGTTATTGTTAACCTTTTTGTCTCCAATTGTCGTCATAACGATGAGGTGTTGCATTTTTGGAAACGAGATTGACATAGAGTCAAAATTTGCTAAAT

*PGK1_P_-*co*ilvD-TEF1_t_*

GCGGCCGCGCCAGAGGTATAGACATAGCCAGACCTACCTAATTGGTGCATCAGGTGGTCATGGCCCTTCCGCGGACTCTTTTCTTCTAACCAAGGGGGTGGTTTAGTTTAGTAGAACCTCGTGAAACTTACATTTACATATATATAAACTTGCATAAATTGGTCAATGCAAGAAATACATATTTGGTCTTTTCTAATTCGTAGTTTTTCAAGTTCTTAGATGCTTTCTTTTTCTCTTTTTTACAGATCATCAAGGAAGTAATTATCTACTTTTTACAACAAATATAAAACAAATGGAATTCAAATATAACGGAAAAGTTGAATCAGTGGAACTCAATAAATATTCTAAGACATTGACTCAAGATCCAACACAACCAGCGACTCAAGCGATGTACTACGGCATTGGTTTTAAAGATGAGGATTTCAAAAAAGCTCAGGTCGGAATCGTCAGCATGGATTGGGACGGAAATCCATGTAATATGCACTTGGGAACACTTGGGAGTAAAATCAAAAGTTCTGTCAACCAAACTGACGGATTGATTGGACTTCAATTTCATACTATTGGAGTTTCTGATGGAATTGCTAACGGAAAGCTTGGCATGAGATATTCTTTGGTCAGTCGTGAAGTGATTGCTGACAGCATCGAAACCAACGCTGGCGCAGAATATTATGATGCCATCGTTGCCATTCCCGGTTGTGATAAAAATATGCCAGGGTCAATTATCGGAATGGCTCGCTTAAATCGTCCGTCAATTATGGTCTATGGTGGAACGATTGAACATGGCGAATATAAAGGTGAAAAATTAAATATTGTTTCGGCCTTTGAATCTCTGGGACAAAAAATCACTGGAAATATTTCTGATGAAGATTATCATGGCGTTATTTGCAATGCCATTCCAGGGCAAGGTGCTTGCGGAGGAATGTACACTGCTAATACCCTGGCTGCCGCTATTGAAACTTTAGGAATGAGTTTACCTTATTCCTCTTCCAATCCAGCAGTCAGTCAAGAAAAACAAGAAGAGTGTGATGAAATTGGTTTAGCCATCAAAAATTTATTAGAAAAAGATATTAAACCAAGTGATATCATGACCAAAGAAGCTTTTGAAAATGCCATAACAATTGTCATGGTCCTTGGAGGCTCAACCAATGCTGTGCTTCATATCATTGCAATGGCAAATGCCATAGGTGTAGAAATTACGCAAGATGATTTCCAACGTATTTCAGATATTACCCCTGTTCTTGGCGATTTCAAACCGAGCGGAAAATATATGATGGAAGATCTGCACAAAATTGGTGGCCTTCCTGCTGTTTTGAAATACCTACTTAAAGAAGGAAAACTTCACGGTGATTGTTTGACCGTCACAGGTAAAACTTTGGCTGAAAATGTTGAAACAGCATTAGATTTGGACTTTGACAGCCAAGATATTATGCGACCACTAAAAAATCCAATTAAAGCTACTGGACATTTACAAATCTTGTACGGTAATCTTGCCCAAGGGGGTTCTGTTGCAAAAATTTCTGGTAAAGAAGGCGAATTTTTCAAAGGAACTGCTCGTGTTTTTGACGGAGAACAACACTTTATCGATGGCATTGAGTCTGGCCGATTGCATGCCGGTGATGTTGCGGTCATTAGAAATATTGGCCCAGTCGGAGGTCCGGGAATGCCAGAGATGTTAAAACCAACCTCAGCATTAATTGGAGCAGGACTTGGAAAATCTTGTGCCCTAATTACTGACGGAAGATTTTCTGGCGGCACACACGGCTTTGTTGTGGGTCATATCGTCCCTGAAGCAGTTGAAGGTGGGTTGATTGGTTTAGTTGAAGATGATGATATTATCGAAATTGATGCGGTGAATAATAGTATTAGTTTAAAAGTTTCTGATGAAGAAATTGCTAAACGACGTGCCAATTATCAAAAACCAACCCCTAAAGCAACGCGTGGTGTCCTTGCAAAATTTGCCAAACTTACACGCCCCGCTAGTGAAGGTTGCGTTACAGATTTATAGGACAATAAAAAGATTCTTGTTTTCAAGAACTTGTCATTTGTATAGTTTTTTTATATTGTAGTTGTTCTATTTTAATCAAATGTTAGCGTGATTTATATTTTTTTTCGCCTCGACATCATCTGCCCAGATGCGAAGTTAAGTGCGCAGAAAGTAATATCATGCGTCAATCGTATGTGAATGCTGGTCGCTATACTGCCGCGGTATTCACGTAGACGGATAGGTATAGCCAGACATCAGCAGCATACTTCGGGAACCGTAGGCGTCGAC

*TEF1_P_-*co*ilvN^M13^-CYC1_t_*

GACATGGAGGCCCAGAATACCCTCCTTGACAGTCTTGACGTGCGCAGCTCAGGGGCATGATGTGACTGTCGCCCGTACATTTAGCCCATACATCCCCATGTATAATCATTTGCATCCATACATTTTGATGGCCGCACGGCGCGAAGCAAAAATTACGGCTCCTCGCTGCAGACCTGCGAGCAGGGAAACGCTCCCCTCACAGACGCGTTGAATTGTCCCCACGCCGCGCCCCTGTAGAGAAATATAAAAGGTTAGGATTTGCCACTGAGGTTCTTCTTTCATATACTTCCTTTTAAAATCTTGCTAGGATACAGTTCTCACATCACATCCGAACATAAACAACCATGGCTAACTCTGACGTTACTAGACACATCTTGTCTGTTTTGGTTCAAGACGTTGACGACGACTTCTCTAGAGTTTCTGGTATGTTCACTAGAAGAGCTTTCAACTTGGTTTCTTTGGTTTCTGCTAAGACTGAAACTCACGGTATCAACAGAATCACTGTTGTTGTTGACGCTGACGAATTGAACATCGAACAAATCACTAAGCAATTGAACAAGTTGATCCCAGTTTTGAAGGTTGTTAGATTGGACGAAGAAACTACTATCGCTAGAGCTATCATGTTGGTTAAGGTTTCTGCTGACTCTACTAACAGACCACAAATCGTTGACGCTGCTAACATCTTCAGAGCTAGAGTTGTTGACGTTGCTCCAGACTCTGTTGTTATCGAATCTACTGGTACTCCAGGTAAGTTGAGAGCTTTGTTGGACGTTATGGAACCATTCGGTATCAGAGAATTGATCCAATCTGGTCAAATCGCTTTGAACAGAGGTCCAAAGACTATGGCTCCAGCTAAGATCGACCACGTTATCTCTTACGTTACTTTGACTTTCACTCCATCTTCTCACATCAGATCTAACAGAAAGGGTAGATCTACAACCTGAAGTCTAGGTCCCTATTTATTTTTTTTAATAGTTATGTTAGTATTAAGAACGTTATTTATATTTCAAATTTTTCTTTTTTTTCTGTACAAACGCGTGTACGCATGTAACATTATACTGAAAACCTTGCTTGAGAAGGTTTTGGGACGCTCGAAGGCTTTAATTTGCG
